# LIN28B-mediated PI3K/AKT pathway activation promotes metastasis in colorectal cancer models

**DOI:** 10.1172/JCI186035

**Published:** 2025-01-14

**Authors:** Alice E. Shin, Kensuke Sugiura, Secunda W. Kariuki, David A. Cohen, Samuel P. Flashner, Andres J. Klein-Szanto, Noriyuki Nishiwaki, Dechokyab De, Neil Vasan, Joel T. Gabre, Christopher J. Lengner, Peter A. Sims, Anil K. Rustgi

**Affiliations:** 1 Division of Digestive and Liver Diseases, Department of Medicine, and; 2 Department of Surgery, Herbert Irving Comprehensive Cancer Center, Vagelos College of Physicians and Surgeons; Columbia University Irving Medical Center, New York, New York, USA.; 3 Histopathology Facility, Fox Chase Cancer Center, Philadelphia, Pennsylvania, USA.; 4 Division of Hematology and Oncology, Department of Medicine, Herbert Irving Comprehensive Cancer Center, Vagelos College of Physicians and Surgeons, Columbia University Irving Medical Center, New York, New York, USA.; 5 Department of Biomedical Sciences, School of Veterinary Medicine, and Institute for Regenerative Medicine, University of Pennsylvania, Philadelphia, Pennsylvania, USA.; 6 Department of Systems Biology, Herbert Irving Comprehensive Cancer Center, Vagelos College of Physicians and Surgeons, Columbia University Irving Medical Center, New York, New York, USA.

**Keywords:** Gastroenterology, Oncology, Colorectal cancer, Mouse models, Oncogenes

## Abstract

Colorectal cancer (CRC) remains a leading cause of cancer death because of metastatic spread. LIN28B is overexpressed in 30% of CRCs and promotes metastasis, yet its mechanisms remain unclear. In this study, we genetically modified CRC cell lines to overexpress LIN28B, resulting in enhanced PI3K/AKT pathway activation and liver metastasis in mice. We developed genetically modified mouse models with constitutively active *Pik3ca* that form intestinal tumors progressing to liver metastases with an intact immune system, addressing the limitations of previous *Pik3ca*-mutant models, including long tumor latency, mixed histology, and lack of distant metastases. The PI3Kα-specific inhibitor alpelisib reduced migration and invasion in vitro and metastasis in vivo. We present a comprehensive analysis of vertical inhibition of the PI3K/AKT pathway in CRC using the FDA-approved drugs alpelisib and capivasertib (an AKT inhibitor) in combination with LY2584702 (a ribosomal protein S6 kinase inhibitor) in CRC cell lines and mouse- and patient-derived organoids. Tissue microarrays from patients with CRC verified that LIN28B and PI3K/AKT pathway activation correlate with CRC progression. These findings highlight the critical role of the LIN28B-mediated PI3K/AKT pathway in CRC metastasis, the therapeutic potential of targeted inhibition, and the promise of patient-derived organoids in precision medicine in metastatic CRC.

## Abstract 2

LIN28B overexpression activates the PI3K/AKT pathway in colorectal cancer, promoting metastasis. Combined vertical inhibition with PI3K pathway-targeting drugs reduces tumor spread.

## Graphical abstract

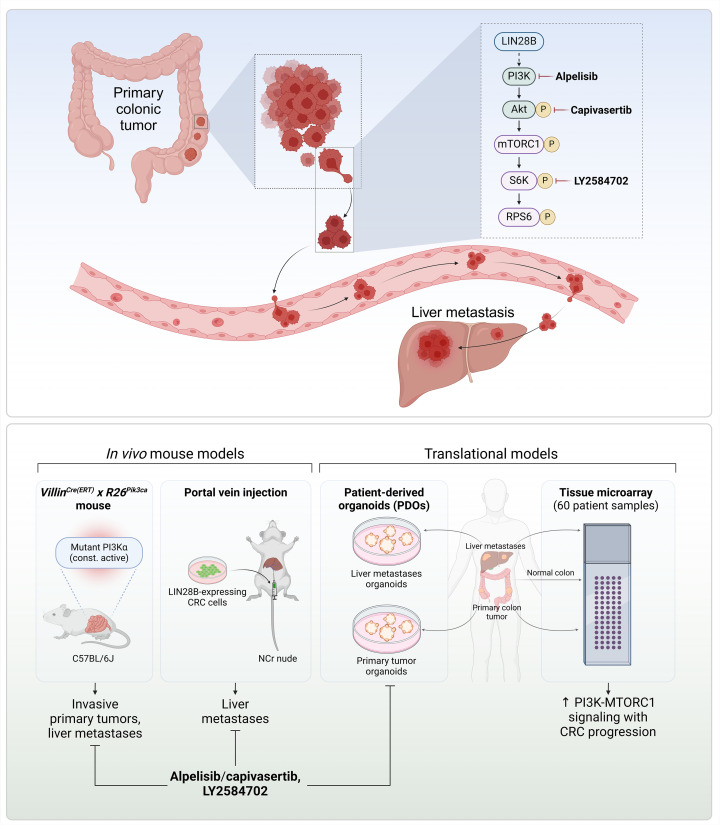

## Introduction

Colorectal cancer (CRC) remains a substantial public health concern in the United States and worldwide. With 1.9 million new cases globally in 2022 and an estimated 150,000 new cases in the United States in 2024, CRC is the third most common cancer in the world (1). Localized CRC benefits from effective therapies and has a 5-year survival rate of up to 91%. However, metastatic CRC (mCRC) has a dismal prognosis, with a 5-year survival rate of 13% (2). Thus, there is a compelling rationale to unravel the molecular mechanisms underlying mCRC to foster the integration of translational therapeutics.

Classically, CRC has served as a model for understanding the cooperation of oncogenic mutations (e.g., *KRAS*, *BRAF*, *PIK3CA*, and *LIN28B*) and the inactivation of tumor suppressor genes (e.g., *APC*, *TP53*, and *SMAD4*) in fostering primary tumorigenesis (3). Among these, the role of LIN28B has garnered attention as an RNA-binding protein influencing gene regulation and cancer progression. The LIN28 proteins (LIN28A and LIN28B) regulate gene expression by binding to mRNA posttranscriptionally. The tumor-suppressing microRNA *let-7* is the most well-characterized target of LIN28, but we and others have demonstrated both *let-7*–dependent and –independent regulation of LIN28 (4–10). While both LIN28A and LIN28B paralogs are critical to various human developmental processes, LIN28B has emerged as a potent oncogene across several cancers (11). LIN28B is overexpressed in esophageal, breast, and prostate cancers and is often an indicator of advanced disease state and poor prognosis (4, 11–13). In CRC, LIN28B is overexpressed in 30% of cases and is associated with poor survival rates and an increased probability of tumor recurrence (12). Additionally, LIN28B overexpression promotes CRC initiation, progression, and metastasis (4, 5, 12, 14). Despite LIN28B’s clear role in inducing tumorigenesis and metastasis, the exact mechanisms through which it exerts these effects remain elusive.

The phosphatidylinositol-3-kinase (PI3K) family of enzymes mediate signals downstream of cell membrane receptors, such as receptor tyrosine kinases (e.g., epidermal growth factor receptor [EGFR] and insulin receptors) and G protein–coupled receptors (15). Class I PI3Ks consist of one catalytic subunit with 4 isoforms (p110α, p110β, p110γ, and p110δ) that most commonly associate with the p85 regulatory subunit. The resulting heterodimers are termed PI3Kα, PI3Kβ, PI3Kγ, or PI3Kδ, after their respective catalytic subunit (16). Activation of PI3K facilitates downstream signaling primarily through protein kinase B (PKB, or AKT) (15). AKT then activates downstream targets to regulate cell survival, proliferation, differentiation, and metabolism (17, 18). Hyperactivation of class I PI3Ks promotes aberrant cell growth and malignant transformation (15, 19). Additionally, PI3K activation has been suggested to promote metastasis, likely as a result of its role in epithelial-mesenchymal transition and angiogenesis (20–23).

In CRC, PI3K pathway mutations occur in approximately 50%–70% of cases, with alterations in the *PIK3CA* gene present in 15%–20% of CRC cases, making *PIK3CA* one of the most commonly mutated genes in CRC (24–26). These mutations are typically associated with poor clinical outcomes and reduced efficacy of anti-EGFR monoclonal antibody therapies (27–30). Despite the prevalence of *PIK3CA* mutations, there are currently no US Food and Drug Administration–approved (FDA-approved) therapies targeting *PIK3CA*-mutant mCRC. Furthermore, PI3K inhibitors have shown low response rates as monotherapy in patients with *PIK3CA*-mutant and widely mCRC (31, 32), underscoring the need for more effective combination therapeutic strategies. Vertical inhibition of the PI3K pathway at multiple points (upstream and downstream) using FDA-approved drugs is a promising approach, analogous to the successful BRAF/MEK inhibition in *BRAF*-mutant cancers (33). The recent development of PI3Kα-specific inhibitors, which are less toxic and more specific, enhances the feasibility and effectiveness of combination therapies (34, 35).

Currently, the primary therapeutic regimen for mCRC includes systemic chemotherapy and targeted therapies that focus on pathways such as EGFR, angiogenesis, and multi-kinase inhibitors. While effective, these traditional chemotherapeutic drugs are DNA-damaging agents and thus affect all rapidly dividing cells, leading to toxicity and limiting their duration. Existing targeted therapies, although more specific, also face challenges such as resistance, toxicity, and limited efficacy in some patients. Given the lethality of mCRC, it is crucial to investigate the mechanisms of metastasis and develop targeted therapies (36).

In this study, we demonstrate that LIN28B expression in CRC cells activates the PI3K/AKT pathway and promotes metastasis to the liver. We developed genetically engineered mouse models with mutant *Pik3ca* that form primary intestinal tumors within 3 months, with a subset progressing to liver metastasis, overcoming the limitations of previous models (35, 36). Additionally, we provide a comprehensive analysis of vertical inhibition of PI3Kα, AKT, and ribosomal protein S6 kinase (S6K) using FDA-approved drugs, including alpelisib and capivasertib, in combination with LY2584702, in CRC cell lines and 3D patient-derived organoids (PDOs). Treatment with these inhibitors effectively reduced cell proliferation, migration, and invasion in 2D cell lines, organoid growth in 3D organoids, and liver metastasis formation in vivo. Furthermore, our study demonstrates that PDOs can advance precision medicine in mCRC, as drug responses were dependent on mutational profiles obtained from clinical testing conducted on tumor tissues and whole-exome sequencing (WES) of PDOs. Our findings underscore the critical role of the PI3K/AKT pathway in CRC metastasis and highlight the therapeutic potential of targeting this pathway to manage mCRC.

## Results

### LIN28B expression in CRC cells activates the PI3K/AKT pathway and promotes liver metastasis.

**Figure 1:**
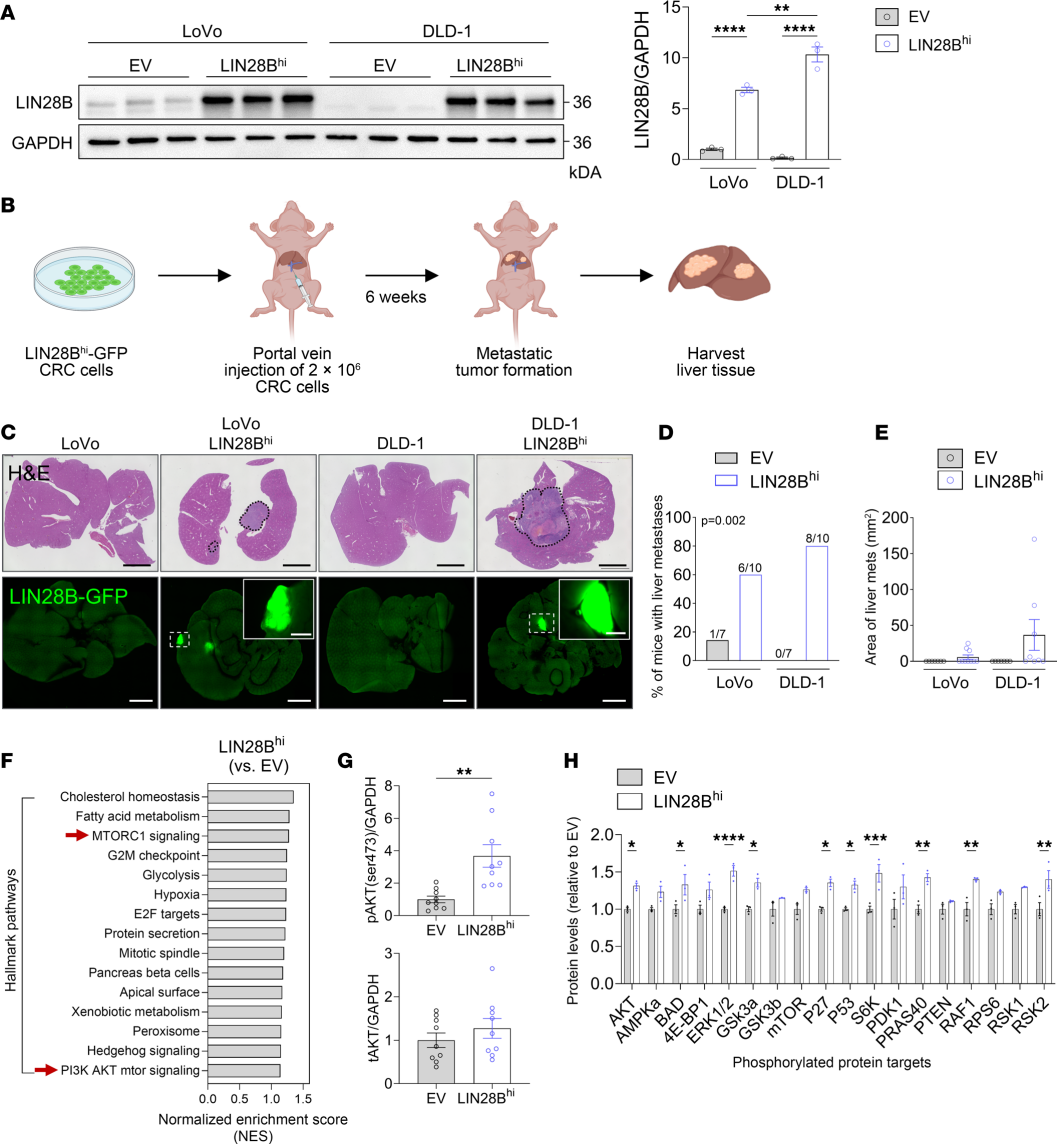
LIN28B expression in CRC cells activates the PI3K/AKT pathway and promotes liver metastasis. (**A**) Western blot of LIN28B protein levels in LoVo and DLD-1 CRC cell lines with either EV or LIN28B overexpression vector (LIN28B^hi^), normalized to GAPDH and LoVo EV (1-way ANOVA, mean ± SEM). (**B**) Experimental setup for in vivo colorectal liver metastasis assay. (**C**) Representative H&E and GFP images of liver sections from mice injected with CRC cells. Scale bars: 5 mm; scale bars for insets: 1 mm. (**D**) Proportion of mice that developed liver metastases (χ^2^ test). (**E**) Quantification of the size of liver metastases in each group (1-way ANOVA, mean ± SEM). (**F**) GSEA showing hallmark pathways enriched in LoVo LIN28B^hi^ cells compared with EV cells (*n* = 3). (**G**) Western blot analysis of p-AKT (Ser473) and t-AKT in CRC cells (2-tailed Student’s unpaired *t* test, mean ± SEM). (**H**) Quantification of phosphorylated protein targets involved in the PI3K/AKT pathway in LIN28B^hi^ cells relative to EV cells as measured by AKT pathway phosphorylation array (2-tailed Student’s unpaired *t* test between EV and LIN28B^hi^ for each protein, mean ± SEM). *P < 0.05, **P < 0.01, ***P < 0.001, ****P < 0.0001.

To determine whether LIN28B expression in CRC cells leads to metastasis formation, we generated CRC cells with genetic modification of LIN28B expression as described previously (5, 14). Endogenous LIN28B levels are low in human LoVo and DLD-1 CRC cell lines, which correspondingly exhibit minimal metastatic propensity when injected into the portal vein of immune-compromised mice. Thus, we generated LoVo and DLD-1 cells with LIN28B expression and GFP fluorescence (LIN28B^hi^). The increase in LIN28B protein levels was verified via immunoblotting (Figure 1A). These LIN28B^hi^ CRC cells were then injected into the portal vein of 6- to 8-week-old Taconic NCr nude mice (*CrTac NCr-Foxn1^nu^*), and liver tissues were harvested 6 weeks after injection (Figure 1B). As anticipated, injection of parental LoVo and DLD-1 cells containing empty vectors (EV) resulted in minimal metastatic formation in the liver, with metastases forming in 1 of 7 mice (14%) for LoVo cells and none in DLD-1 cells (0 of 7 mice). Conversely, injections of LoVo LIN28B^hi^ and DLD-1 LIN28B^hi^ cells led to significantly higher rates of liver metastasis, with metastases occurring in 6 of 10 (60%) and 8 of 10 (80%) mice, respectively (Figure 1, C–E). We verified that the increased metastatic propensity of LIN28B^hi^ cells was not attributable to increased growth or prolonged survival of the 2D cell lines (Supplemental Figure 1A; supplemental material available online with this article; https://doi.org/10.1172/JCI186035DS1). These results reveal that LIN28B expression in CRC cells enhances their metastatic potential.

To elucidate the downstream signaling pathways activated by LIN28B, we conducted RNA sequencing (RNA-Seq) of LIN28B^hi^ cells compared with EV cells. Subsequent gene set enrichment analysis (GSEA) revealed that MTORC1 signaling and PI3K AKT MTOR signaling hallmark pathways were upregulated in LIN28B^hi^ cells (Figure 1F). This was verified by Kyoto Encyclopedia of Genes and Genomes (KEGG) analysis of upregulated genes in LIN28B^hi^ LoVo and DLD-1 cells compared with their respective EV control cells. By overlapping of the upregulated genes between the 2 cell lines, 2,061 common genes were identified and analyzed using KEGG. The colorectal cancer pathway was among the significant hits, which included genes involved in the PI3K/AKT pathway (Supplemental Figure 1B). WES further revealed an increased number of mutations in genes within the PI3K/AKT pathway in LIN28B^hi^ cells when compared with EV cells (Supplemental Figure 1C).

To confirm the RNA-Seq results, we performed immunoblotting to detect phosphorylated AKT (p-AKT) (Ser473) levels, a key effector of PI3K/AKT pathway activation. Consistent with our sequencing data, LIN28B^hi^ cells exhibited increased p-AKT levels compared with EV controls, with no changes in total AKT (t-AKT) (Figure 1G). A comprehensive analysis using a PI3K/AKT pathway phosphorylation array showed that LIN28B^hi^ cells harbored elevated phosphorylation of several critical proteins within the pathway, including AKT, Bcl-2–associated death promoter (BAD), extracellular signal–regulated kinase 1 and 2 (ERK1/2), glycogen synthase kinase 3-α (GSK3α), p27, p53, S6K, proline-rich Akt substrate of 40 kDa (PRAS40), RAF1, and ribosomal S6 kinase 2 (RSK2) (Figure 1H). Taken together, these data suggest that LIN28B expression in CRC cells activates the PI3K/AKT pathway with concurrent promotion of liver metastasis.

### Activation of the PI3K/AKT pathway induces colonic crypt hyperplasia and drives CRC tumorigenesis and metastasis.

To validate our hypothesis that the PI3K/AKT pathway acts downstream of LIN28B, we aimed to replicate the metastatic propensity of LIN28B^hi^ cells by activating the PI3K/AKT pathway pharmacologically using SC79, a pan-AKT activator. Increasing the concentration of SC79 to 20 μM or higher compromised cell viability in DLD-1 cells, thereby preventing the collection of high-quality proteins for further analysis (Supplemental Figure 2A). This increased sensitivity in DLD-1 cells, which may be attributed to existing *PIK3CA* mutations (unlike in *PIK3CA*-wild-type LoVo cells), guided our decision to use 5 μM SC79 for subsequent assays. Immunoblotting verified that 5 μM SC79 increased p-AKT and phosphorylated ribosomal protein S6 (p-RPS6; downstream of S6K) levels in both LoVo and DLD-1 EV cells (Supplemental Figure 2B). Treatment with 5 μM SC79 increased cell migration as observed in the wound healing (scratch) assay (Supplemental Figure 2C) and enhanced invasion capabilities as measured by the QCM ECMatrix Cell Invasion Assay (EMD Millipore), evaluating the ability of tumor cells to invade through an extracellular matrix (ECM) model (Supplemental Figure 2D).

**Figure 2:**
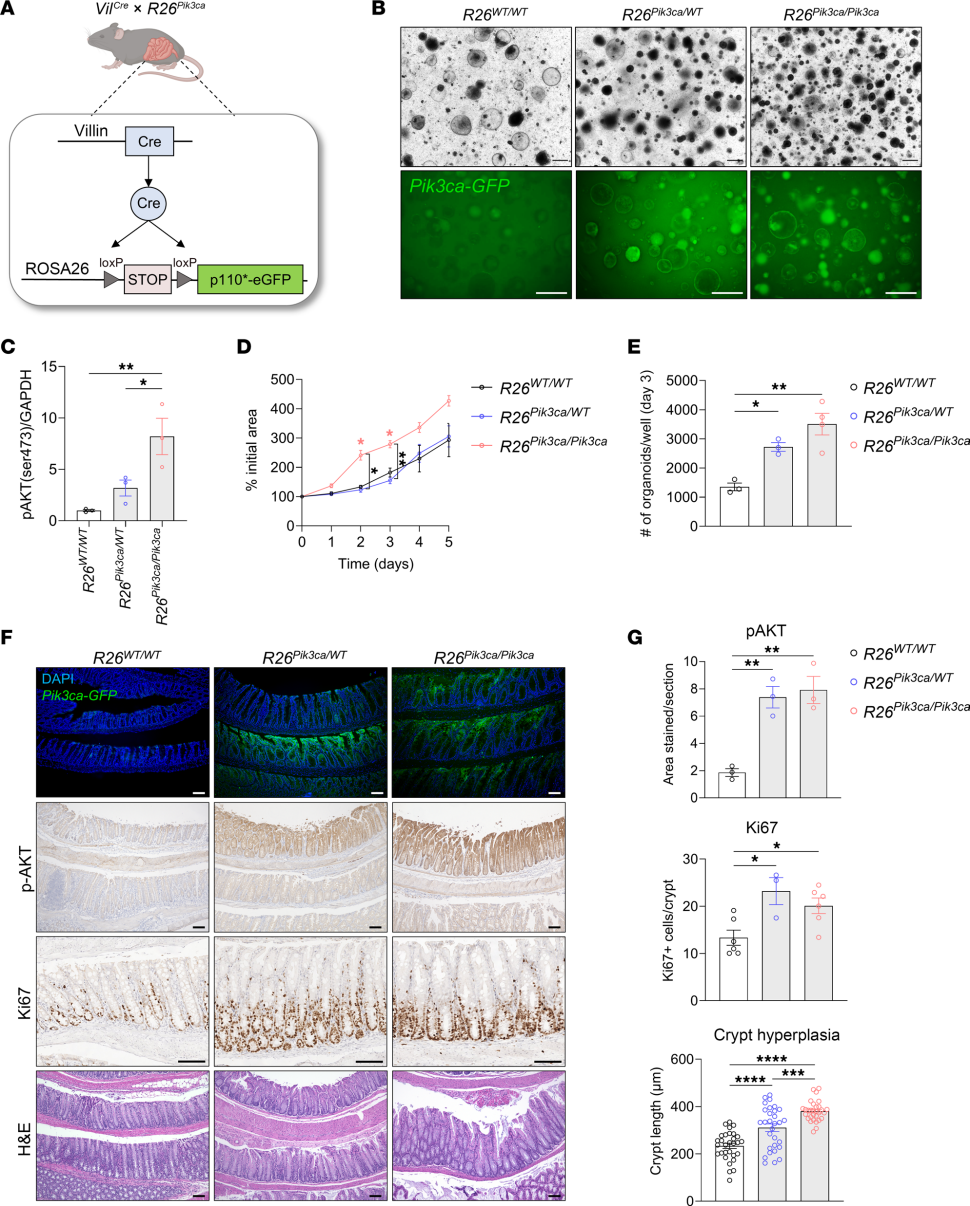
Genetic activation of the PI3K/AKT pathway enhances organoid growth ex vivo and induces colonic crypt hyperplasia in vivo. (**A**) Schematic representation of the genetic cross to generate *Vil^Cre^ R26^Pik3ca^* mice on a C57BL/6J background. (**B**) Representative bright-field and GFP images of colonic organoids derived from *R26^WT/WT^*, *R26^Pik3ca/WT^*, and *R26^Pik3ca/Pik3ca^* mice cultured for 5 days. Scale bars: 500 μm (*n* = 3). (**C**) Immunoblot quantification of p-AKT (Ser473) levels relative to GAPDH in colonic organoids derived from *R26^WT/WT^*, *R26^Pik3ca/WT^*, and *R26^Pik3ca/Pik3ca^* mice, normalized to *R26^WT/WT^* (*n* = 3; 1-way ANOVA, mean ± SEM). (**D**) Quantification of growth of colonic organoids from *R26^WT/WT^*, *R26^Pik3ca/WT^*, and *R26^Pik3ca/Pik3ca^* mice, showing percentage increase in initial area (*n* = 3 and 4; 2-way ANOVA, mean ± SEM). (**E**) Quantification of the number of colonic organoids per well on day 3 of culture (1-way ANOVA, mean ± SEM). (**F**) Representative immunofluorescence and IHC images of colonic tissues from *R26^WT/WT^*, *R26^Pik3ca/WT^*, and *R26^Pik3ca/Pik3ca^* mice, showing *Pik3ca*-GFP, p-AKT (Ser473), Ki67, and H&E staining. Scale bars: 100 μm. (**G**) Quantification of p-AKT–stained area per section, Ki67-positive cells per crypt, and crypt length in colonic tissues from *R26^WT/WT^*, *R26^Pik3ca/WT^*, and *R26^Pik3ca/Pik3ca^* mice. Crypt length was measured every 100 μm along the length of the colon (1-way ANOVA, mean ± SEM). *P < 0.05, **P < 0.01, ***P < 0.001, ****P < 0.0001.

We next aimed to independently corroborate the metastatic propensity of LIN28B^hi^ cells by genetically activating the PI3K/AKT pathway. To achieve this, we generated a *Villin^Cre^ Rosa26^Pik3ca^* (*Vil^cre^* *R26^Pik3ca^*) mouse model on a C57BL/6J background. This genetic configuration allows for the induced expression of a constitutively active mouse catalytic p110α subunit of PI3Kα and eGFP in all intestinal and colonic epithelial cells, starting at embryonic day 12.5 (Figure 2A) (37, 38). Colonic crypts were isolated from these mice to culture 3D colonic organoids. Analysis included 3 genotypes: wild-type (*R26^WT/WT^*), heterozygous mutant (*R26^Pik3ca/WT^*), and homozygous mutant (*R26^Pik3ca/Pik3ca^*), with all groups being hemizygous for *Vil^Cre^*. The mutant organoids were verified as GFP positive (Figure 2B) and exhibited increased p-AKT levels (Figure 2C). Homozygous mutant organoids demonstrated an increased growth rate (Figure 2D), and both heterozygous and homozygous mutant organoids showed enhanced organoid formation efficiency, as determined by quantification of the number of organoids formed from an equivalent number of plated crypts on day 3 (Figure 2, B and E). In vivo analyses of the distal to proximal end of the colon showed both GFP expression and elevated p-AKT levels in the colonic epithelium of heterozygous and homozygous mutant mice (Figure 2, F and G, and Supplemental Figure 2E). Interestingly, these groups also exhibited an increased number of cells expressing the marker of proliferation (Ki67) and heightened crypt hyperplasia, marked by increased crypt lengths measured along the distal to proximal colon, indicative of augmented proliferation (Figure 2, F and G, and Supplemental Figure 2E).

**Figure 3:**
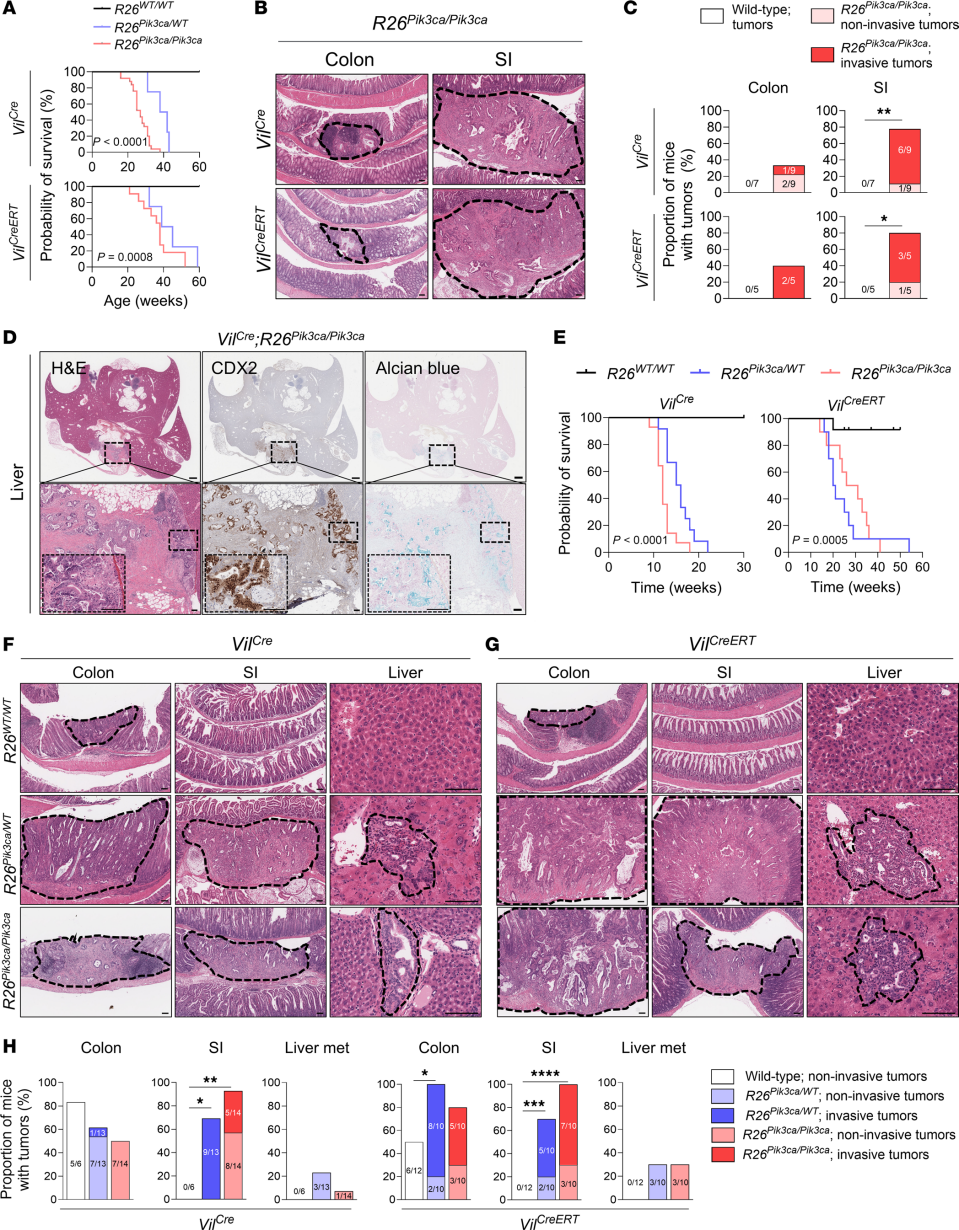
Genetic activation of the PI3K/AKT pathway promotes tumorigenesis, tumor invasiveness, and liver metastasis in a mouse model of CRC. (**A**) Kaplan-Meier survival curves of *Vil^Cre^* and *Vil^CreERT2^* mice with either *R26*^WT/WT^, *R26^Pik3ca/WT^*, or *R26^Pik3ca/Pik3ca^* genotype (*n* = 7, 4, and 25 for *Vil^Cre^*, *n* = 5, 4, and 11 for *Vil^CreERT^*; log-rank test). (**B**) Representative H&E images of the colon and SI tissues from mice with *R26^Pik3ca/Pik3ca^* genotype. Dashed lines outline the tumors. Scale bars: 100 μm. (**C**) Proportion of mice with noninvasive adenomas and invasive adenocarcinomas in the colon and SI from *Vil^Cre^* and *Vil^CreERT2^* mice with either *R26*^WT/WT^ or *R26^Pik3ca/Pik3ca^* genotype (Fisher’s exact test). (**D**) Representative H&E, CDX2, and Alcian blue staining of a liver metastasis from a 28-week-old *Vil^Cre^ R26^Pik3ca/Pik3ca^* mouse. Scale bars: 1 mm; scale bars for insets: 100 μm. (**E**) Kaplan-Meier survival curves of *Vil^Cre^* and *Vil^CreERT2^* mice treated with AOM and tamoxifen (*n* = 8–14 for *Vil^Cre^*, *n* = 10–12 for *Vil^CreERT^*; log-rank test). (**F** and **G**) Representative H&E images of colon, SI, and liver tissues from *Vil^Cre^* (**F**) and *Vil^CreERT2^* (**G**) mice treated with AOM. Dashed lines outline the tumors. Scale bars: 100 μm. (**H**) Proportion of mice with noninvasive adenomas and invasive adenocarcinomas in the colon and SI, and liver metastases from mice treated with AOM (χ^2^ test between *R26*^WT/WT^ and either *R26^Pik3ca/WT^* or *R26^Pik3ca/Pik3ca^* genotype). *P < 0.05, **P < 0.01, ***P < 0.001, ****P < 0.0001.

Longitudinal studies revealed that while *Vil^Cre^ R26^WT/WT^* mice remained healthy at 60 weeks of age, *Vil^Cre^ R26^Pik3ca/WT^* mice succumbed to tumors between 31 and 43 weeks of age, and *Vil^Cre^ R26^Pik3ca/Pik3ca^* mice succumbed to tumors between 16 and 38 weeks of age (Figure 3A). We first verified that the mice were not dying due to altered glucose metabolism, considering that the activation of the PI3K/AKT pathway promotes glucose uptake in cells (39). A glucose tolerance test revealed no significant difference between *Vil^Cre^ R26^WT/WT^* and *Vil^Cre^ R26^Pik3ca/Pik3ca^* mice (Supplemental Figure 3A). *Vil^Cre^ R26^Pik3ca/Pik3ca^* mice exhibited a spectrum of neoplastic lesions. In the colon, well-differentiated adenomas confined to the mucosa were observed in 2 of 9 mice (22%), and moderately differentiated cancers that penetrated the basement membrane were observed in 1 of 9 mice (11%) (Figure 3, B–D, and Supplemental Figure 3B). In the small intestine (SI), tumors were present in 7 of 9 mice (78%), with well-differentiated adenomas in 1 mouse (11%) and moderately differentiated adenocarcinomas in 6 of 9 mice (67%) (Figure 3, B–D, and Supplemental Figure 3B). Additionally, liver metastases were verified in 2 of 9 mice (22%) that also had intestinal adenocarcinomas, as shown by staining with CDX2 (a marker of intestinal epithelial cells) and Alcian blue (which highlights mucin production) (Figure 3D) (14, 40). This observation was verified in *Vil^CreERT^ R26^Pik3ca/Pik3ca^* mice treated with tamoxifen at 6 weeks of age, which is an inducible model for temporal regulation of mutant *Pik3ca* expression (41). Two of five mice (40%) developed moderately differentiated colonic cancers, 3 of 5 mice (60%) developed moderately differentiated SI cancers, and 1 of 5 mice (20%) developed a well-differentiated SI adenoma. However, these mice did not have liver metastases by 21–40 weeks of age (Figure 3, A–C, and Supplemental Figure 3B).

To further explore the effects of PI3Kα activation on colorectal metastasis, we used a well-established carcinogen-induced sporadic mouse model of CRC. Injections of 10 mg/kg azoxymethane (AOM) every week for 6 weeks have been reported to lead to well-differentiated colonic adenomas that remain confined to the basement membrane in wild-type C57BL/6J mice, with minimal effects on the SI or the liver (42–44). *Vil^Cre^ R26^Pik3ca^* mice were injected with AOM, and tissues from *R26^WT/WT^* mice were harvested at 30 weeks after the first injection of AOM for analysis (Supplemental Figure 3C). *R26^Pik3ca/WT^* and *R26^Pik3ca/Pik3ca^* mice had to be euthanized when they exhibited signs of severe illness, such as substantial weight loss or a severely deteriorated condition (Figure 3E and Supplemental Figure 3, C and D). Administering tamoxifen at 9 weeks after the first injection of AOM to *Vil^CreERT^ R26^Pik3ca^* mice enabled temporal control of mutant PI3Kα expression after primary colonic tumor formation, allowing focused analysis of the effects of active PI3Kα on metastatic progression (Supplemental Figure 3C). Survival curves highlight reduced lifespans in both heterozygous and homozygous *R26^Pik3ca^* mutant mice using either *Vil^Cre^* or *Vil^CreERT^* alleles (Figure 3E). Histologic assessments revealed well-differentiated colonic adenomas in *R26^WT/WT^* mice treated with AOM, with adenomas detected in 5 of 6 (83%) *Vil^Cre^* and 6 of 12 (50%) *Vil^CreERT^* mice. By contrast, a subset of *R26^Pik3ca^* mutant mice developed moderately differentiated colonic adenocarcinomas (Figure 3, F–H, and Supplemental Figure 3, E and F). Additionally, the majority of *R26^Pik3ca^* mutant mice developed SI adenocarcinomas localized primarily in the duodenum and jejunum (Figure 3, F–H, and Supplemental Figure 3, E and F). Remarkably, *R26^Pik3ca^* mutant mice developed metastases in the liver, as observed in 4 of 27 (14.8%) *Vil^Cre^* and 6 of 20 (30%) *Vil^CreERT^* mice (Figure 3, F–H, and Supplemental Figure 3, E and F). We verified that the liver metastases originated from primary intestinal tumors by CDX2 staining (Supplemental Figure 3G). It is conceivable that penetrance of primary tumors and liver metastasis would be greater if mice lived longer, especially in the case of *Vil^Cre^ R26^Pik3ca^* mice; however, this was mitigated by deteriorated condition of the mice that prompted euthanasia at the specified time points, most likely due to tumor-induced obstruction. Other organs, including the lung, pancreas, and thymus, remained unaffected, suggesting metastatic tropism to the liver. Collectively, our data demonstrate that genetic activation of the PI3K/AKT pathway promotes primary tumorigenesis and liver metastasis in our mouse models.

### Alpelisib impairs LIN28B-induced cell migration and invasion and inhibits PI3Kα-induced organoid growth.

Having established the role of PI3Kα activation in CRC metastasis in vivo, we next assessed the therapeutic potential of inhibiting PI3Kα to inhibit metastatic progression. To date, such a therapeutic approach has not been pursued for FDA approval, affording new perspectives in mCRC (36). For this purpose, we used alpelisib, a PI3Kα-specific inhibitor currently approved by the FDA for treating hormone receptor–positive (*HR*-positive), human epidermal growth factor receptor 2–negative (*HER2*-negative), *PIK3CA*-mutated advanced or metastatic breast cancer (45).

**Figure 4:**
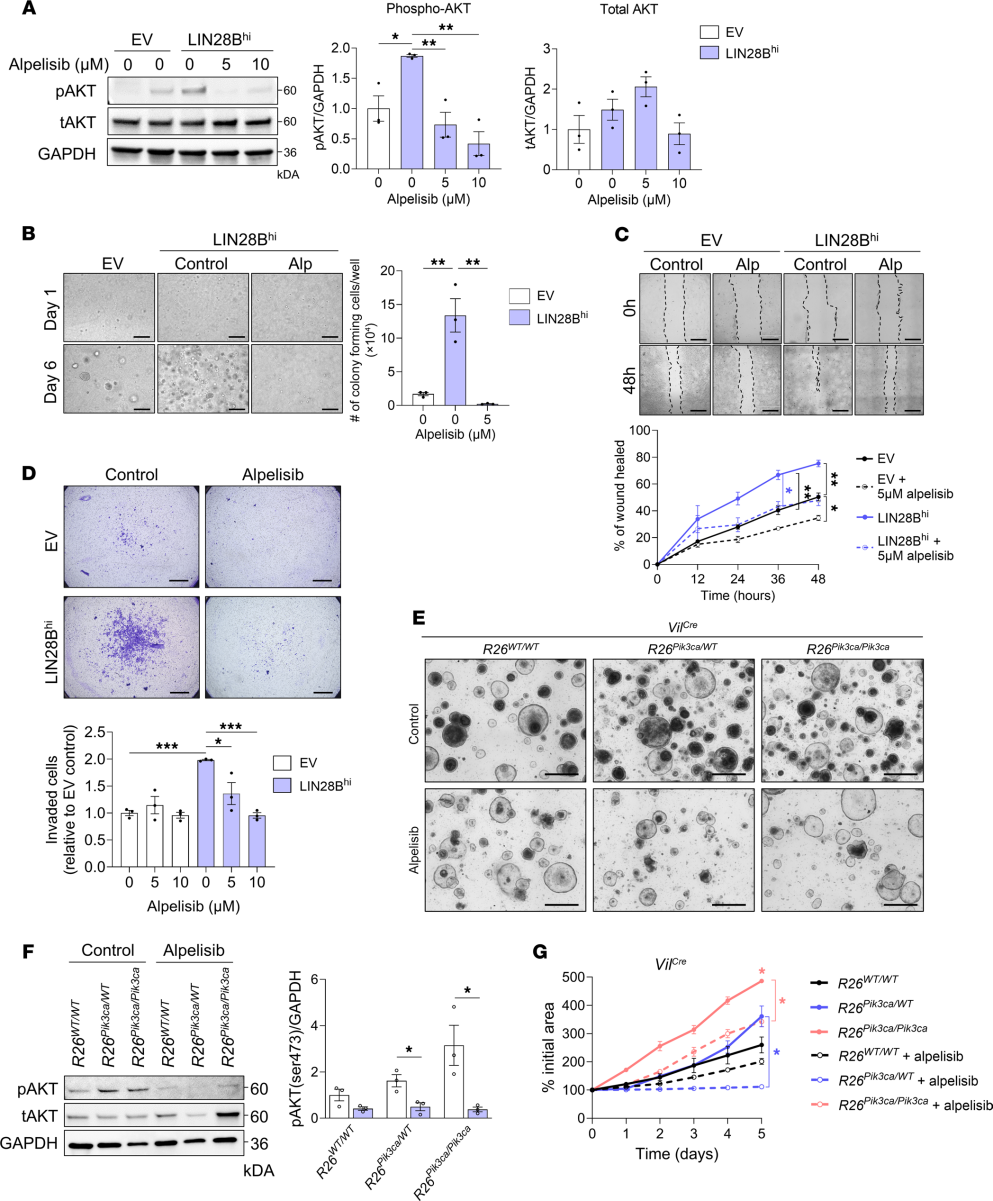
Alpelisib impairs LIN28B-induced cell migration and invasion and inhibits PI3Kα-induced organoid growth. (**A**) Western blot analysis of p-AKT (Ser473) and t-AKT in CRC cells (1-way ANOVA, mean ± SEM). (**B**) Colony formation assay of LIN28B^hi^ CRC cells treated with 5 μM alpelisib. Scale bars: 500 μm (1-way ANOVA, mean ± SEM). (**C**) Wound healing assay showing cell migration of CRC cells treated with 5 μM alpelisib at 0 hours. Scale bars: 500 μm (*n* = 4; 2-way ANOVA, mean ± SEM). (**D**) Transwell ECM invasion assay of CRC cells treated with 5 or 10 μM alpelisib. Scale bars: 1 mm (1-way ANOVA, mean ± SEM). (**E**) Representative bright-field images of colonic organoids derived from *Vil^Cre^* mice treated with 5 μM alpelisib every 2 days for 5 days. Scale bars: 500 μm. (**F**) Western blot analysis of LIN28B, p-AKT (Ser473), and t-AKT in colonic organoids derived from *Vil^Cre^* mice treated with 5 μM alpelisib (2-tailed Student’s unpaired *t* test within each group, mean ± SEM). (**G**) Quantification of growth of colonic organoids derived from *Vil^Cre^* mice treated with 5 μM alpelisib (*n* = 3–5; 2-way ANOVA, mean ± SEM; significance for day 5 is shown with asterisks; the asterisk above the data point signifies significance when compared with the *R26^WT/WT^* control group). *P < 0.05, **P < 0.01, ***P < 0.001.

A viability assay revealed that cell viability began to decrease at concentrations starting from 10 μM of alpelisib in LoVo and DLD-1 cell lines but did not decrease at 5 μM (Supplemental Figure 4, A and B). Immunoblot analysis showed that LIN28B^hi^ cells exhibited elevated p-AKT levels compared with EV cells, and treatment with 5 μM and 10 μM alpelisib reduced p-AKT levels in LIN28B^hi^ cells to levels comparable to those in EV cells, indicating effective pathway inhibition (Figure 4A). Based on this, we selected 5 μM for subsequent experiments, as this concentration does not reduce the viability of any of the cell lines used (Supplemental Figure 4C). A soft agar colony formation assay, which assesses anchorage-independent growth, revealed that LIN28B^hi^ cells treated with 5 μM alpelisib exhibited reduced colony formation, reverting to control levels observed in EV cells (Figure 4B). A wound healing assay revealed that treatment with 5 μM alpelisib reduced cell migration at 36 and 48 hours after treatment in LIN28B^hi^ cells, with a notable effect also observed in EV cells at 48 hours (Figure 4C). The QCM ECMatrix Cell Invasion Assay showed that alpelisib had no effect on EV cells but reduced the number of invading LIN28B^hi^ cells at both 5 μM and 10 μM, demonstrating alpelisib’s potent anti-invasion effects (Figure 4D).

We next tested the effects of alpelisib using colonic organoids derived from *Vil^Cre^ R26^Pik3ca^* mice (Figure 4E). Organoids from all 3 genotypes (*R26^WT/WT^*, *R26^Pik3ca/WT^*, and *R26^Pik3ca/Pik3ca^*) were treated with 5 μM alpelisib. Immunoblotting demonstrated decreased p-AKT (Ser473) in *R26^Pik3ca/WT^* and *R26^Pik3ca/Pik3ca^* organoids, corroborating the inhibitor’s efficacy (Figure 4F). Alpelisib significantly reduced organoid growth in both *R26^Pik3ca/WT^* and *R26^Pik3ca/Pik3ca^* organoids, with no discernible effect on *R26^WT/WT^* organoids (Figure 4G). These results indicate that alpelisib impairs LIN28B-induced cell proliferation, migration, and invasion and inhibits PI3Kα-induced organoid growth.

### Alpelisib inhibits colorectal liver metastasis formation in mice.

**Figure 5:**
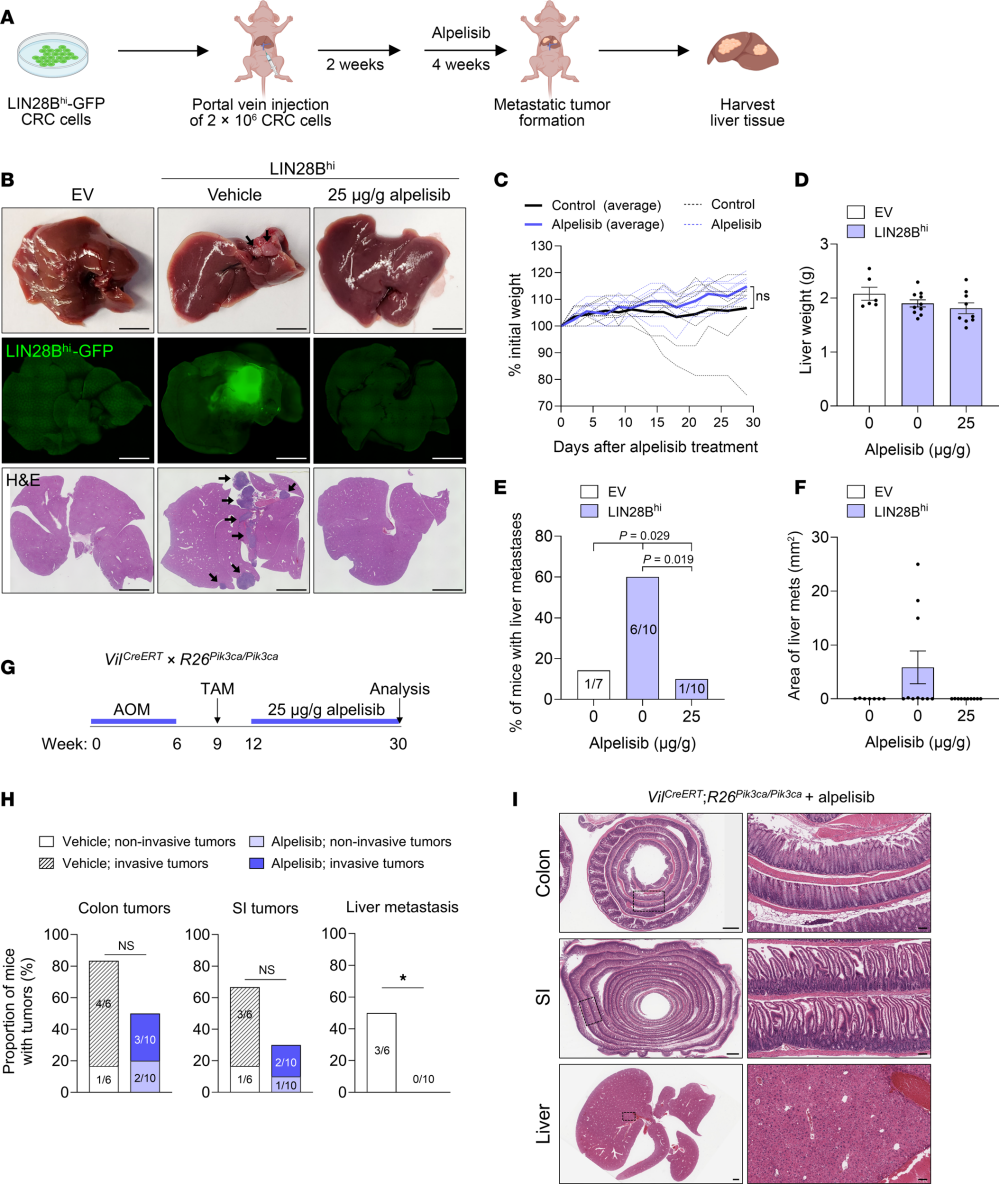
Alpelisib inhibits colorectal liver metastasis formation in mice. (**A**) Experimental setup for investigating the effect of alpelisib on colorectal liver metastasis formation. (**B**) Representative images of liver tissues from mice injected with CRC cells and treated with vehicle or alpelisib. Gross liver morphology with black arrows denoting liver metastases (top), GFP fluorescence indicating liver metastases from LIN28B^hi^-GFP CRC cells (middle), and H&E staining with black arrows denoting liver metastases (bottom) are shown. Scale bars: 5 mm. (**C**) Weight change of mice over the course of the experiment, expressed as percentage of initial weight. Dashed lines, individual mice; solid line, average of all mice in group (*n* = 7 and 10). (**D**) Quantification of liver weight (1-way ANOVA, mean ± SEM). (**E**) Proportion of mice with liver metastases in each group (χ^2^ test). The dataset for the control groups in this graph is the same as the data reported in Figure 1D. (**F**) Quantification of the area of liver metastases in each group (1-way ANOVA, mean ± SEM). (**G**) Experimental setup in which *Vil^CreERT^ R26^Pik3ca/Pik3ca^* mice were treated with AOM to induce tumor formation, followed by tamoxifen, and subsequently treated with 25 μg/g alpelisib after primary tumors had formed. (**H**) Proportion of *Vil^CreERT^ R26^Pik3ca/Pik3ca^* mice with tumors in the colon and SI and liver metastases (Fisher’s exact test). (**I**) Representative H&E-stained images of colon, SI, and liver tissues from *Vil^CreERT^ R26^Pik3ca/Pik3ca^* mice treated with alpelisib. Scale bars: 1 mm; scale bars for insets: 100 μm. *P < 0.05.

To investigate the in vivo effects of alpelisib on CRC metastasis, we used the mCRC portal vein injection model. LIN28B^hi^ CRC cells were injected into the portal vein of NCr nude mice. Two weeks after injection, mice were given oral gavage of 25 μg/g alpelisib every 2 days for a period of 4 weeks, after which the livers were harvested for analysis (Figure 5, A and B) (46). Mice treated with alpelisib appeared healthier and exhibited less weight loss compared with the vehicle-treated control group, suggesting improved general health, although the difference was not statistically significant owing to variability within the vehicle-treated group (Figure 5C). No changes were observed in liver weight, indicating that alpelisib did not adversely affect liver mass (Figure 5D). Treatment with alpelisib resulted in a significant reduction of liver metastases derived from LIN28B^hi^ CRC cells, with only 1 in 10 alpelisib-treated mice (10%) developing a micrometastasis, highlighting the efficacy of alpelisib in inhibiting metastatic progression (Figure 5, B, E, and F).

To confirm these findings, we used the transgenic *Vil^CreERT^ R26^Pik3ca/Pik3ca^* model of mCRC. These mice were treated with AOM and tamoxifen, followed by 25 μg/g alpelisib administration every 2 days (Figure 5G). Alpelisib treatment inhibited liver metastasis formation, as observed by gross inspection and verified through histologic analysis of serial liver sections (Figure 5, H and I). These findings underscore the potential of alpelisib to inhibit liver metastasis formation in 2 independent mouse models of mCRC.

### Pharmacologic inhibition of the S6K/RPS6 axis suppresses LIN28B-driven cell migration and invasion in CRC cells.

**Figure 6:**
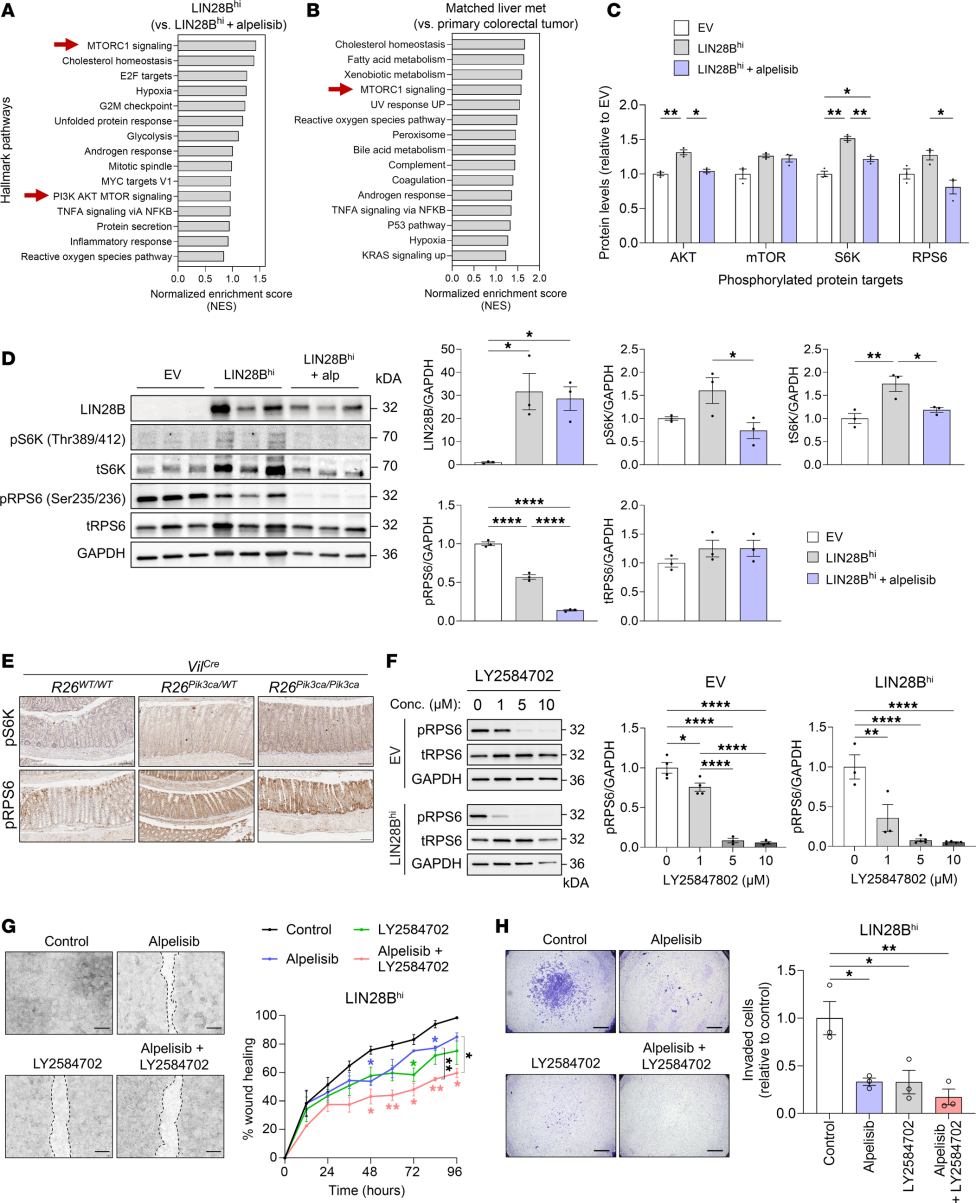
Pharmacologic inhibition of the S6K/RPS6 axis suppresses LIN28B-driven cell migration and invasion in CRC cells. (**A**) GSEA from RNA-Seq showing hallmark pathways enriched in LIN28B^hi^ cells compared with LIN28B^hi^ cells treated with 5 μM alpelisib. (**B**) GSEA from RNA-Seq showing hallmark pathways enriched in liver metastasis compared with matched primary tumors in patients with CRC (GSE50760). (**C**) Quantification of phosphorylated protein targets involved in the PI3K/AKT pathway in EV, LIN28B^hi^, and LIN28B^hi^ cells treated with 5 μM alpelisib (1-way ANOVA for each protein target, mean ± SEM). (**D**) Western blot analysis of LIN28B, p-AKT (Ser473), p-S6K (Thr389/412), total S6K, p-RPS6 (Ser235/236), t-RPS6, and GAPDH in CRC cells treated with 5 μM alpelisib (alp, alpelisib; 1-way ANOVA, mean ± SEM). (**E**) Representative IHC images of p-S6K (Thr389/412) and p-RPS6 (Ser235/236) in colonic tissues from *Vil^Cre^* mice. Scale bars: 100 μm. (**F**) Western blot analysis of p-RPS6, t-RPS6, and GAPDH in CRC cells treated with varying concentrations of LY2584702 (S6K inhibitor) (1-way ANOVA, mean ± SEM). (**G**) Wound healing assay showing cell migration of LIN28B^hi^ CRC cells (*n* = 3; 2-way ANOVA, mean ± SEM). Scale bars: 500 μm. (**H**) Transwell ECM invasion assay of LIN28B^hi^ CRC cells (1-way ANOVA, mean ± SEM). Scale bars: 1 mm. *P < 0.05, **P < 0.01, ****P < 0.0001.

To elucidate the downstream effects of PI3Kα inhibition by alpelisib, we conducted RNA-Seq of LIN28B^hi^ CRC cells, untreated and treated with alpelisib. GSEA identified MTORC1 signaling as a top hallmark pathway that was enriched in vehicle-treated cells compared with alpelisib-treated cells (Figure 6A). Consistent with this finding, we analyzed a published dataset (NCBI Gene Expression Omnibus [GEO] GSE50760) that involved RNA-Seq of samples from primary CRC and matched liver metastases from 18 patients with CRC (47). “MTORC1 signaling” was enriched in the matched liver metastases relative to primary tumors (Figure 6B), prompting us to further investigate MTORC1 signaling downstream of the PI3K/AKT pathway with and without alpelisib treatment.

The PI3K/AKT pathway phosphorylation array showed that levels of p-AKT, p-S6K (downstream of MTORC1), and p-RPS6 (downstream of S6K) in LIN28B^hi^ cells treated with alpelisib were reduced to levels comparable to those in EV cells (Figure 6C), suggesting that while LIN28B expression increases MTORC1 signaling, alpelisib can effectively reverse this effect. However, mTOR phosphorylation was not significantly reduced by alpelisib treatment, likely because of PI3K-independent mechanisms regulating mTOR activation (48, 49). This observation was corroborated by immunoblotting, which showed decreased levels of p-S6K and p-RPS6 upon alpelisib treatment in LIN28B^hi^ CRC cells (Figure 6D). Staining of the colonic tissues from *Vil^Cre^ R26^Pik3ca^* mice verified increased staining of p-S6K and p-RPS6 in both *R26^Pik3ca/WT^* and *R26^Pik3ca/Pik3ca^* mouse models (Figure 6E and Supplemental Figure 5).

Given these findings, we explored the potential additive effects of inhibiting S6K activation in combination with alpelisib. LY2584702, a selective ATP-competitive S6K inhibitor, was tested at concentrations of 1, 5, and 10 μM on both EV and LIN28B^hi^ CRC cells. Concentrations of 1, 5, and 10 μM of LY2584702 reduced p-RPS6 levels in EV and LIN28B^hi^ cells without inducing cytotoxicity (Figure 6F and Supplemental Figure 4, A and B). A wound healing assay demonstrated that either 5 μM alpelisib or 5 μM LY2584702 reduced cell migration in LIN28B^hi^ cells at 48 or 72 hours, respectively, when compared with the vehicle-treated control group (Figure 6G). When LY2584702 was combined with alpelisib, there was a greater effect in reducing wound healing, indicating an additive effect (Figure 6G). The QCM ECMatrix Cell Invasion Assay revealed a decreased ability of LIN28B^hi^ CRC cells to invade through the ECM with treatments of alpelisib or LY2584702. The combination treatment with alpelisib and LY2584702, although not to a statistically significant degree in comparison with single-treatment groups, showed a trend toward decreased invasion (Figure 6H). Collectively, these results demonstrate that pharmacologic inhibition of the S6K/RPS6 axis using LY2584702 suppresses LIN28B-driven cell migration and invasion in CRC cells, and combining PI3Kα inhibition with S6K inhibition may have an additive effect.

### Pharmacologic inhibition of PI3Kα and S6K impairs the growth of patient-derived CRC organoids.

Next, we established 3D PDOs from primary CRC tumors (Table 1). Surgical specimens of colonic tumors were collected, and cells were isolated and cultured in Matrigel. Notably, the organoids designated as CRC28 were derived from a patient whose liver metastasis was resected concurrently, providing a unique opportunity to establish a matched liver metastasis organoid line (“CRC28met”). Histologic examination verified the tumor status (differentiation and stage) of each sample, alongside its adjacent normal colon tissue and liver metastasis in the case of CRC28 (Supplemental Figure 6A). Before organoid culture, tumor samples were analyzed for mutations in genes known to impact clinical management via the Columbia Solid Tumor Panel (CSTP) specific for colorectal and pancreatic cancers (Table 2). Interestingly, 4 of 5 samples (80%) with *PIK3CA* mutations exhibited concurrent *KRAS* mutations, while the remaining sample had a *BRAF* mutation. To understand this observation further, we analyzed data from The Cancer Genome Atlas (TCGA) Pan-Cancer Atlas, which verified a significant co-occurrence of *KRAS* and *PIK3CA* mutations in CRC (Supplemental Figure 6B).

**Figure 7:**
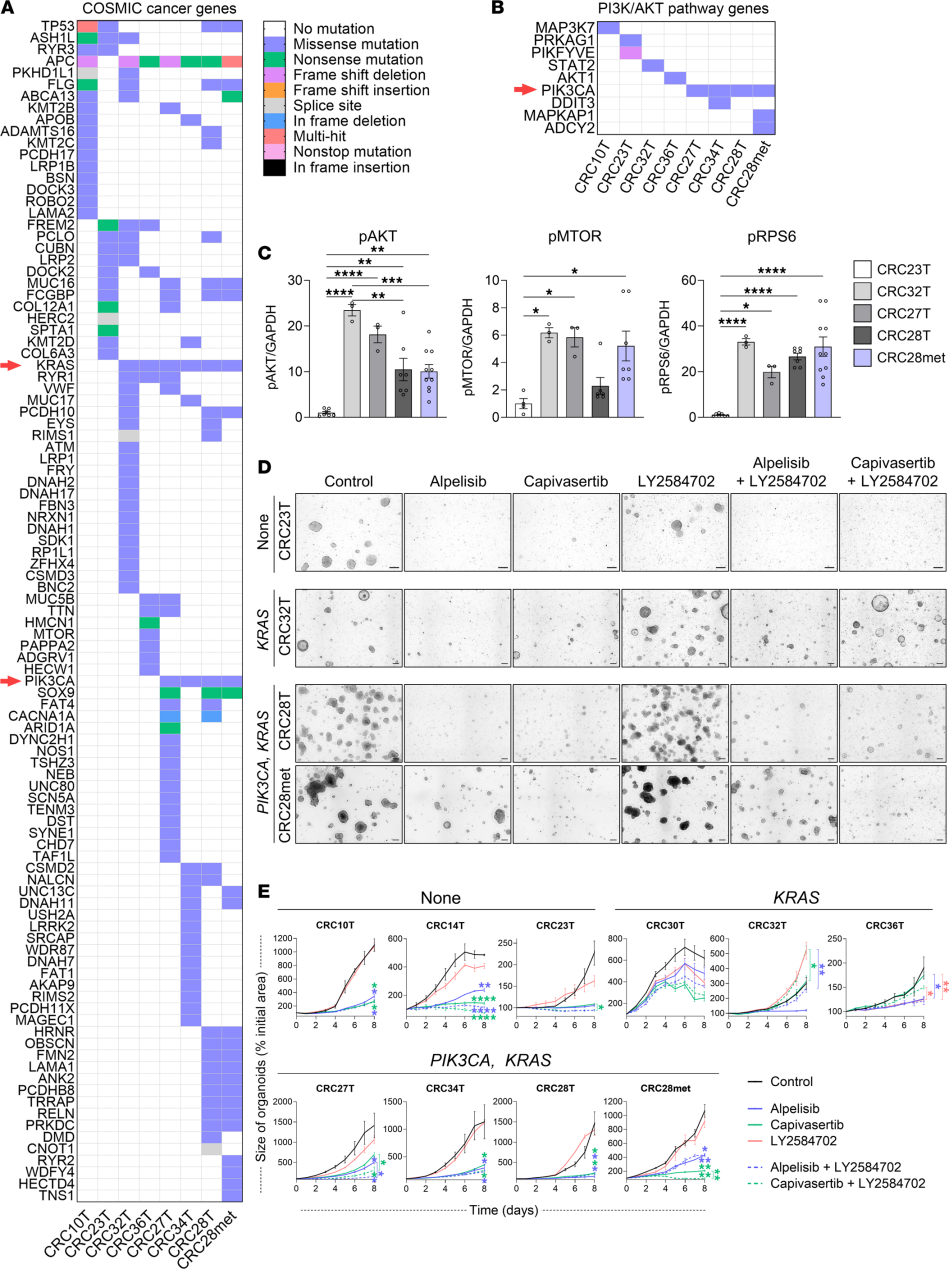
Pharmacologic inhibition of PI3Kα and S6K impairs the growth of CRC PDOs. (**A**) Heatmap showing top 200 CRC mutations in Catalogue of Somatic Mutations in Cancer (COSMIC) cancer genes in PDO lines identified by WES. Only genes with mutations are shown. Mutation types are color coded as indicated in the legend (T, primary tumor; met, liver metastasis). (**B**) Heatmap showing mutations in PI3K/AKT pathway genes in PDO lines identified by WES. Refer to the list of PI3K/AKT pathway genes used in Supplemental Methods. Only genes with mutations are shown. (**C**) Quantification of Western blot analysis of p-AKT (Ser473), phosphorylated mTOR (p-MTOR) (Ser2448), and p-RPS6 (Ser235/236) in PDO lines (1-way ANOVA, mean ± SEM). (**D** and **E**) Representative bright-field images (**D**) and growth curves (**E**) of PDOs treated with the inhibitors every other day for 8 days. Scale bars: 100 μm (*n* = 3; 2-way ANOVA, mean ± SEM). *P < 0.05, **P < 0.01, ***P < 0.001, ****P < 0.0001.

After PDO establishment, WES was performed to verify mutations identified by the CSTP and to discover potential additional mutations. Each organoid line displayed unique mutational profiles (Figure 7A). Interestingly, we observed mutations in one or more genes of the PI3K/AKT pathway in each organoid line (Figure 7B). Specifically, CRC27T, CRC34T, CRC28T, and CRC28met harbored missense mutations in the *PIK3CA* gene (Figure 7B). Additionally, CRC28T and CRC28met displayed distinct mutational profiles. The differences between CRC28T and CRC28met were further analyzed using g:Profiler (https://biit.cs.ut.ee/gprofiler/gost) for biological pathway enrichment (KEGG, Reactome, WikiPathways) (Supplemental Figure 6, C and D). Immunoblotting for p-AKT, p-MTOR, and p-RPS6 demonstrated elevated pathway activity in PDO lines with *PIK3CA* and/or *KRAS* mutations (Figure 7C).

We next evaluated the efficacy of targeted therapeutic agents in these models. The PDOs were treated with alpelisib and LY2584702 to dissect the functional consequences of PI3Kα and S6K inhibition in CRC. To validate these findings and explore the broader clinical applicability of PI3K/AKT pathway inhibition, we included capivasertib, a pan-AKT inhibitor recently approved by the FDA for use in *HR*-positive, *HER2*-negative locally advanced or metastatic breast cancer with *PIK3CA*, *AKT1*, or *PTEN* mutations (50). PDOs with no mutations in clinically actionable genes according to the CSTP (CRC10T, CRC14T, CRC23T) were highly sensitive to alpelisib or capivasertib as monotherapy. In these lines, LY2584702 alone did not significantly affect organoid growth. However, in the CRC14T and CRC23T lines, the combination of either alpelisib or capivasertib with LY2584702 enhanced the suppression of organoid growth (Figure 7, D and E, and Supplemental Figure 7). For organoids with *KRAS* mutations (CRC30T, CRC32T, CRC36T), the effects of the treatments varied and did not exhibit consistent patterns. Alpelisib effectively suppressed growth in CRC32T and CRC36T. However, in CRC32T, LY2584702 paradoxically increased growth, and its combination with alpelisib neutralized alpelisib’s effect. Capivasertib was ineffective in both lines. CRC30T showed no significant response to any treatment (Figure 7, D and E, and Supplemental Figure 7). Organoids with both *PIK3CA* and *KRAS* mutations (CRC27T, CRC34T, CRC28T, CRC28met) were highly sensitive to either alpelisib or capivasertib as monotherapy (Figure 7, D and E, and Supplemental Figure 7). This suggests that the presence of a *PIK3CA* mutation makes organoids more amenable to targeted treatments compared with having a *KRAS* mutation alone. In CRC27T and CRC28met organoid lines, the combination with LY2584702 further enhanced the suppression of organoid growth (Figure 7, D and E, and Supplemental Figure 7). The growth-suppressing effects of alpelisib or capivasertib in combination with LY2584702 were verified in organoids derived from the colonic tumors of *Vil^Cre^ R26^Pik3ca/Pik3ca^* mice (Supplemental Figures 8 and 9). Collectively, the ability to pharmacologically inhibit the PI3K/AKT pathway in *PIK3CA*-mutant PDOs underscores the potential of these inhibitors in combination therapies for primary and mCRC, especially in tumors with concurrent *PIK3CA* and *KRAS* mutations.

### PI3K/S6K signaling correlates with disease progression in CRC patient samples.

**Figure 8:**
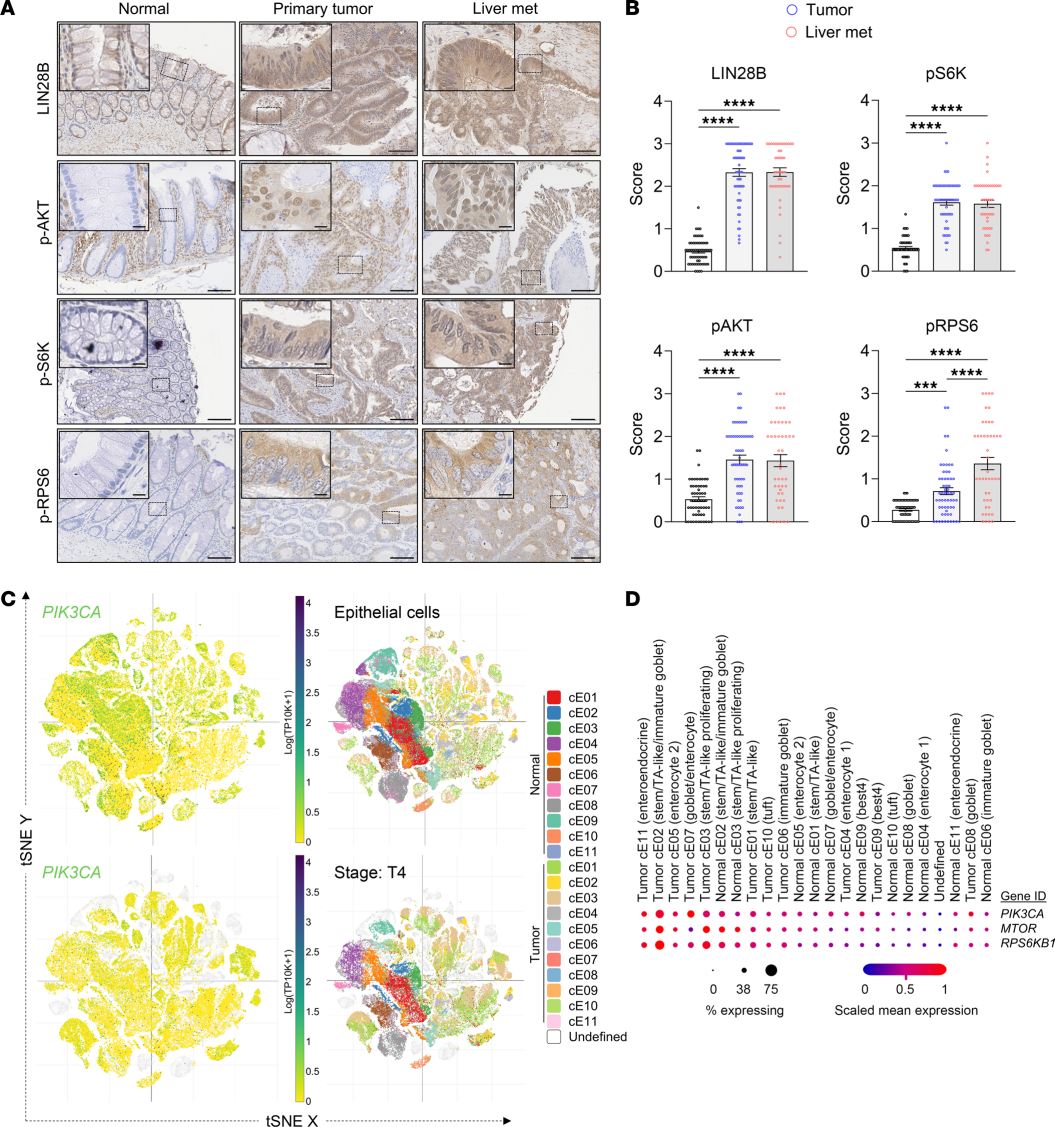
PI3K/S6K signaling correlates with disease progression in CRC patient samples. (**A**) Representative IHC images of LIN28B, p-AKT (Ser473), p-S6K (Thr389/412), and p-RPS6 (Ser235/236) in normal adjacent colon tissue, primary colon tumor, and liver metastases from 60 patients with CRC. Scale bars: 100 μm; scale bars for insets: 10 μm. (**B**) Quantification of IHC staining scores for LIN28B, p-AKT, p-S6K, and p-RPS6 (*n* = 60; 1-way ANOVA, mean ± SEM). (**C**) T-distributed stochastic neighbor embedding (t-SNE) plots showing the expression of *PIK3CA* in all epithelial cells (top) and in T4 stage tumor cells (bottom) from the Human Colon Cancer Atlas single-cell sequencing dataset (c295) comprising 371,223 cells. (**D**) Dot plot showing scaled mean expression and percentage of cells expressing *PIK3CA*, *MTOR*, and *RPS6KB1* across different cell clusters (normal colonic epithelial and tumor cells) identified in the Human Colon Cancer Atlas dataset. cE, colonic epithelium. ***P < 0.001, ****P < 0.0001.

To validate our experimental findings and their relevance to clinical progression, we constructed a tissue microarray (TMA) from samples obtained from 60 patients with CRC. Each TMA core included tissue from adjacent normal colonic tissue, primary colonic tumors, and liver metastases from the same patients. IHC analysis revealed that 100% of primary colonic tumors and liver metastases were positively stained for LIN28B (Figure 8, A and B, and Supplemental Figure 10). We previously reported that 30% of CRCs express LIN28B (12); this discrepancy is likely because the TMAs were constructed from patients who had already developed liver metastases. Additionally, elevated p-AKT and p-S6K levels were observed in both the primary CRC and matched liver metastases when compared with the adjacent normal tissues (Figure 8, A and B, and Supplemental Figure 10). Interestingly, the expression of p-RPS6, a downstream effector of both AKT and S6K, was increased in primary tumors compared with adjacent normal tissues, and further elevated in liver metastases compared with the primary tumors (Figure 8, A and B, and Supplemental Figure 10). These findings are supported by single-cell RNA sequencing (scRNA-Seq) data retrieved from the Human Colon Cancer Atlas (c295), which reveals higher expression of *PIK3CA*, *MTOR*, and *RPS6KB1* in tumor cells compared with healthy cells (Figure 8, C and D, and Supplemental Figure 11). Notably, within the tumor cell population, stem/transit-amplifying-like cells show enhanced levels of these genes (Figure 8, C and D, and Supplemental Figure 11). Taken together, the data from TMA IHC and scRNA-Seq suggest that the PI3K signaling pathway, particularly marked by the elevation of p-RPS6, correlates with disease progression in CRC patient samples.

## Discussion

In this study, we provide insights into CRC pathogenesis by demonstrating that LIN28B expression in CRC cells activates the PI3K/AKT pathway, enhancing their metastatic potential to the liver. Our findings highlight that this metastatic process is dependent on the activation of the PI3K/AKT pathway within the CRC cells. Pharmacologic and genetic activation of the PI3K/AKT pathway independently corroborated these findings, showing enhanced cell migration, invasion, primary tumorigenesis, and metastasis. Furthermore, we introduce the first genetically engineered mouse model (GEMM) that develops colonic tumors progressing to liver metastases within an intact immune system, driven by a single oncogenic mutation, *Pik3ca*. Treatment with these inhibitors effectively reduced cell proliferation, migration, and invasion in 2D cell lines, organoid growth in 3D organoids, and liver metastasis formation in vivo.

Our study presents a transgenic mouse model that develops primary intestinal tumors and metastasizes to the liver within an intact immune system, driven by a single oncogenic event, *Pik3ca*, in combination with AOM treatment (51). Previously developed GEMMs of mCRC have typically required multiple oncogenic hits or surgical interventions to achieve similar outcomes. For instance, a GEMM of mCRC involved a surgical procedure to limit adeno-cre infection to the distal colon with homozygous *Apc* conditional knockout and heterozygosity for a latent activated allele of *Kras*. This model resulted in liver metastases in 20% of mice within 24 weeks after adeno-cre injection (52). The iKAP mouse model generated by Boutin et al. eliminated the need for surgery by using direct 4-OH-tamoxifen enema to *Vil^CreERT^* mice with *Apc^fl/fl^*, *Tp53^fl/fl^*, and a Tet-inducible *Kras^G12D^* allele. This model displayed metastases to the liver and lung within 6 weeks in 25% of the mice (53). Similar to our approach, others have also combined GEMMs with AOM treatment to induce CRC metastasis in mice. *Vil^Cre^ Trp53^fl/fl^ Akt^E17K^* mice develop invasive tumors and lymph node metastasis (20%–30% incidence) when treated with AOM, with tumors closely resembling human CMS4 subtype profiles (54). Additionally, *Vil^Cre^ Trp53^fl/fl^* mice treated with AOM develop high-grade adenocarcinomas and lymph node metastases (20%–30% incidence) but none to the liver or lungs (55). Another commonly used mCRC model involves the orthotopic injection of CRISPR/Cas9–engineered organoids with CRC driver mutations *Kras^G12D^* and *Trp53^fl/fl^*. However, this model requires dextran sodium sulfate–induced inflammation prior to implantation to promote the development of a metastatic phenotype (56). mCRC has also been generated by orthotopic injection of organoids with mutations in *Apc*, *Trp53*, *Kras^G12D^*, and *Smad4* (57).

In the broader context of PI3K research, our transgenic mouse model addresses limitations observed in existing GEMMs with *Pik3ca* mutations across various cancer types, including breast cancer. Previous GEMMs with *Pik3ca* mutations often exhibit long tumor latency times, sometimes taking more than a year for tumor growth (58). Additionally, these models frequently develop sarcomas rather than adenocarcinomas, the latter of which are the most common *PIK3CA*-mutant tumor type in patients (59). Moreover, existing models have inconsistent tumor formation and lack of metastatic potential and are often generated in immunocompromised mice, limiting the relevance to human disease (60, 61). Our model overcomes these limitations with genetic evidence of primary intestinal tumors within approximately 3 months, with some tumors progressing to liver metastases, all achieved within an intact immune system. The histology of the tumors in our model closely resembles the colon adenocarcinoma phenotype observed in patients, providing a more accurate representation of *PIK3CA*-driven colon cancers. This notable advance allows for a more precise study of the PI3K pathway’s role in tumor progression and metastasis, offering a valuable platform. The ability of our model to generate tumors rapidly and with appropriate histologic characteristics highlights its potential to impact preclinical research and therapeutic development for PI3K-mutant CRC.

The use of PDOs provided a highly relevant model system that recapitulates the genetic, phenotypic, and histologic features of original tumors. Vlachogiannis et al. demonstrated the value of PDOs in predicting clinical outcomes in patients with metastatic pretreated colorectal and gastroesophageal cancers. Their study found that PDOs had a high degree of similarity to patients’ tumors and accurately predicted clinical responses to targeted agents or chemotherapy with a sensitivity of 100%, specificity of 93%, positive predictive value of 88%, and negative predictive value of 100% (62). In our study, PDOs enabled us to evaluate the efficacy of PI3K/AKT pathway inhibitors in a model that closely mimics human CRC, thereby providing insights into potential therapeutic strategies.

WES of PDOs from matched primary tumors and liver metastases revealed 1,070 genes that were mutated in both primary and metastatic organoids. One hundred twenty-two genes were mutated exclusively in the metastatic organoids (11.4%), while 115 genes were mutated exclusively in the primary organoids (10.7%). The majority of mutant genes overlapped between the two, aligning with previous findings that mCRC genomes are not fundamentally different from the genomes of primary CRCs in terms of the mutational landscape or the genes driving tumorigenesis (63–65). Genes mutated in metastases predominantly involve immune suppression, epithelial-mesenchymal transition, and angiogenesis (63), as well as MYC signaling, DNA repair, glycolysis, metabolic processes, and targets of hypoxia-inducible factor (66). Our analysis revealed pathways such as cAMP and MAPK signaling, ECM degradation, GPCR signaling, MMP activation, and VEGFA-VEGFR2 signaling among the genes mutated exclusively in PDOs derived from metastasis. The overlap of mutated genes between primary tumors and metastases suggests that metastatic potential may be predetermined early in tumorigenesis, with metastasis-initiating cells already present among the initial cell clones in the primary tumor (36, 67).

It is notable that 80% of tumor tissues collected to generate PDOs with *PIK3CA* mutations also harbored *KRAS* mutations. This was corroborated by TCGA analysis showing high co-occurrence of *PIK3CA* and *KRAS* mutations in CRC, indicating a synergistic or linked pathway involvement. A study evaluating 504 patients with diverse cancers found that *KRAS* mutations were present in 38% of patients with *PIK3CA* mutations compared with 16% of patients with wild-type *PIK3CA* (*P* = 0.001) (68). Specifically in CRC, the analysis of 83 patients with paired primary tumors and matched metastases revealed that 25% of the tumors with mutant *KRAS* and 4% of wild-type *KRAS* tumors had *PIK3CA* mutations (*P* = 0.008) (69). Furthermore, a study of 655 patients with CRC found that *KRAS* and *PIK3CA* comutations were associated with aggressive clinicopathologic features. Patients with both mutations had poorer overall survival compared with those with only one or neither mutation, emphasizing that the concomitant mutation statuses of *KRAS* and *PIK3CA* should be considered for prognostic evaluations in patients with CRC (70). Collectively, the co-occurrence of *PIK3CA* and *KRAS* has implications for targeted therapies, as treatments targeting *PIK3CA*-mutant CRC must also be effective against tumors harboring both *PIK3CA* and *KRAS* mutations to achieve optimal therapeutic outcomes.

The differential sensitivity for PI3K pathway inhibitors observed in PDOs based on their mutational status underscores the importance of considering mutational profiling when selecting targeted therapies for CRC. Organoids without clinically actionable mutations were highly responsive to alpelisib or capivasertib. By contrast, organoids with *KRAS* mutations exhibited variable treatment responses, highlighting the complexity of targeting this subgroup. Notably, organoids harboring both *PIK3CA* and *KRAS* mutations were consistently more sensitive to these targeted therapies. This suggests that *PIK3CA* mutations could serve as predictive markers for treatment efficacy in *KRAS*-mutant CRCs. This is particularly important given that *KRAS* mutations are present in up to 50% of CRC cases and co-occur with *PIK3CA* mutations (71).

It is important to note that several PI3Kδ inhibitors, such as idelalisib, copanlisib, duvelisib, and umbralisib, have been withdrawn from clinical use by the FDA because of immune-related side effects and, in some trials, a reduction in overall survival (59). In contrast, PI3Kα inhibitors such as alpelisib (45) and inavolisib (72), as well as the AKT inhibitor capivasertib (50), remain in clinical use for specific cancer types, with ongoing efforts to mitigate side effects, such as hyperglycemia, through the development of mutant-selective inhibitors like STX-478 (35) and RLY-2608 (34). Moreover, ongoing clinical trials for alpelisib and capivasertib are exploring their efficacy in various cancers, including CRC. Alpelisib, already FDA approved for *HR*-positive, *HER2*-negative, *PIK3CA*-mutated advanced or metastatic breast cancer (45), is being tested in head and neck squamous cell carcinoma, melanoma, multiple myeloma, gastric cancer, pancreatic cancer, and ovarian cancer. Two clinical trials have investigated alpelisib in CRC. The first was a phase Ib/II multicenter study of encorafenib (BRAF inhibitor) and cetuximab (EGFR inhibitor) or encorafenib, cetuximab, and alpelisib in patients with *BRAF*-mutant mCRC (ClinicalTrials.gov, NCT01719380). This study, completed in October 2015, indicated that the combination therapies with alpelisib were generally well tolerated and recommended further evaluation of their efficacy in CRC treatment. The second phase Ib pharmacokinetics study is an active study assessing the efficacy and safety of alpelisib and capecitabine (chemotherapy) in patients with mCRC who have a *PIK3CA* mutation (NCT04753203). Capivasertib is being investigated in clinical trials for triple-negative breast cancer, B cell non-Hodgkin lymphoma, and prostate cancer. The only ongoing clinical trial examining capivasertib in CRC is the MATCH Screening Trial (NCT02465060), a phase II study evaluating the effectiveness of treatments directed by genetic testing in patients with advanced, refractory solid tumors, lymphomas, or multiple myelomas. Patients with *AKT* mutations will be assigned to capivasertib, while taselisib and copanlisib will target *PIK3CA* or *PTEN* mutant cancers.

The safety profile of LY2584702 has been evaluated in 4 phase I clinical trials, yielding divergent results (73, 74). These studies, however, did not incorporate genetic testing as their selection criteria. Our data using PDOs suggest that LY2584702 could be effective when used in combination with PI3K or AKT inhibitors, particularly in patients with both *PIK3CA* and *KRAS* mutations. Organoids that responded well to LY2584702 combined with either alpelisib or capivasertib were derived either from patients with no clinically actionable CRC-related mutations or from patients with both *KRAS* and *PIK3CA* mutations. Genetic testing and combination therapy could potentially lower the required dose for efficacy to mitigate the toxicity of LY2584702 observed at higher doses. Further studies are needed to explore the efficacy of LY2584702 in combination with PI3K pathway inhibitors and to determine its potential in clinical settings, particularly in patients with specific genetic backgrounds.

This study is highly relevant and timely given the recent advancements in developing PI3K inhibitors that are less toxic and more specific, making combination therapies more feasible. For example, RLY-2608 selectively inhibits mutant PI3Kα, reducing the risk of side effects associated with wild-type PI3Kα inhibition, such as hyperglycemia, rash, or diarrhea. RLY-2608 has shown a partial response and initial antitumor activity at different doses in breast cancer patients, including one who had progressed after 12 prior lines of therapy (34). Another promising example is STX-478, an allosteric, mutant-selective PI3Kα inhibitor that interacts with a previously undescribed allosteric pocket within PI3Kα. STX-478 selectively targets mutant PI3Kα, reducing toxicity and improving efficacy compared with alpelisib. STX-478 avoids metabolic dysfunction, such as hyperglycemia. It has demonstrated robust efficacy in human tumor xenografts, and combining STX-478 with other treatments such as fulvestrant and CDK4/6 inhibitors has provided durable tumor regression without substantial side effects (35).

This study’s impact is highlighted by our investigation into vertical inhibition of the PI3K pathway. Vertical inhibition, which involves targeting both upstream and downstream components of the same pathway, has shown promise in therapeutic interventions, analogous to the successful BRAF/MEK inhibition strategy in BRAF mutant cancers (75–77). Our findings demonstrate the effectiveness of vertical inhibition of the PI3K pathway in *PIK3CA*-mutant CRC, providing a promising approach for more effective and safer therapeutic intervention in mCRC, a field bereft of meaningful impact on 5-year survival rates. It is critical that potential treatments for mCRC pivot on new mechanistic insights, and in this context, we nominate the PI3K pathway as a promising therapeutic target.

Collectively, our study provides insights into the mechanism by which LIN28B mediates CRC metastasis, employing a wide range of models (cell lines, GEMMs, 3D PDOs, and human CRC TMAs) to investigate both the activation and inhibition of the PI3K/AKT pathway downstream of LIN28B. Our findings strongly support the critical role of the PI3K/AKT pathway in CRC metastasis and highlight the therapeutic potential of targeting this pathway.

## Methods

### Sex as a biological variable.

Both female and male mice were used for all mouse experiments to ensure that any sex-based variations in tumor development, progression, and response to intervention were captured. PDOs and TMAs were generated from both male and female patients with CRC. Data were analyzed separately for male and female subjects first to discern any subtle sex-specific differences that may exist; however, no significant differences were observed between male and female subjects.

### Generation of LIN28B^hi^ cell lines.

LoVo and DLD-1 cells were obtained from American Type Culture Collection. LoVo and DLD-1 cells with LIN28B expression were generated using a previously described method (5, 12), as outlined in Supplemental Methods.

### PDO culture.

Tumor tissues were obtained from patients undergoing elective surgery at NewYork-Presbyterian/Columbia University Irving Medical Center with written informed consent under a protocol approved by the Columbia University Institutional Review Board (IRB; protocol AAAT8778). Organoid cultures were prepared as previously described, with minor modifications (78). Note that the protocol used for PDO establishment outlined in Supplemental Methods is also effective for tumor tissues that have been frozen in liquid nitrogen for up to 6 months.

### Portal vein injection of LIN28B^hi^ CRC cells.

All animal studies were approved by the Institutional Animal Care and Use Committee (IACUC) at Columbia University, and all experiments were conducted in compliance with the National Institutes of Health (NIH) guidelines for animal research. Portal vein injection was performed as described previously (5) and is outlined in Supplemental Methods.

### Generation of GEMMs.

*Vil^Cre^ R26^Pik3ca^* mice were produced by mating of B6.Cg-Tg(Vil1-cre)1000Gum/J mice (The Jackson Laboratory, strain 021504) with C57BL/6-Gt(ROSA)26Sortm7(Pik3ca*,EGFP)Rsky/J mice (The Jackson Laboratory, strain 012343). *Vil^CreERT^ R26^Pik3ca^* mice resulted from crossing of B6.Cg-Tg(Vil1-cre/ERT2)23Syr/J mice (The Jackson Laboratory, strain 020282) with strain 012343. Both *Cre* and *CreERT* alleles were maintained in a hemizygous state. Toe clip samples were sent to TransnetYX for genotyping. Colon and SI were washed with cold PBS and collected as Swiss rolls before fixing in 10% neutral-buffered formalin. In addition to genotyping, expression of mutant *Pik3ca* in the intestinal epithelial cells was verified by GFP expression, p-AKT (Ser473) staining, and p-AKT (Ser473) immunoblotting.

### Western blot of cells and organoids.

Immunoblotting was conducted as described in Supplemental Methods using primary antibodies (Supplemental Table 1) and visualized using IRDye secondary antibodies (LI-COR Biosciences 926-68070, 926-32211). Measured protein levels were normalized to either GAPDH or β-actin as endogenous controls.

### PI3K/AKT pathway phosphorylation array.

EV and LIN28B^hi^ LoVo and DLD-1 cells, treated with either vehicle or alpelisib, were sent as frozen cell pellets to RayBiotech for analysis using the Human/Mouse AKT Pathway Phosphorylation Array (RayBiotech AAH-AKT-1).

### Soft agar colony formation assay.

The colony formation assay was performed using the CytoSelect 96-Well In Vitro Tumor Sensitivity Assay (Soft Agar Colony Formation, CBA-150) (Cell Biolabs, Inc.) according to the manufacturer’s instructions.

### Construction of TMA.

A cohort of 60 patients diagnosed with colon carcinoma was selected for the study. The patients’ ages ranged from 44 to 94 years (mean age 68.3 years). Rectal tumors were excluded from the study. The patients were selected based on the availability of primary tumors, normal adjacent colon tissue, and liver metastases. Tissue samples were obtained from patients, and TMAs were constructed by the Molecular Pathology Shared Resource under the Columbia University IRB protocol AAAS3903. Three 2 mm cores of normal adjacent colon mucosa, primary tumor tissue, and liver metastases were collected from each patient and were paraffin-embedded in microarrays. IHC staining was performed on the TMA sections as described in Supplemental Methods.

### Histopathologic analysis.

All pathologic analyses were performed at Histopathology Facility, Fox Chase Cancer Center, in accordance with the consensus report and recommendations for pathologic analysis. The quantitative evaluation of positively stained cells (Ki67, LIN28B, p-AKT, p-S6K, p-RPS6) was performed by manual counting of cells for each sample in a blinded fashion. For IHC analysis, 0 = negative staining involving less than 33% of cells; 1 = weak staining involving 33%–66% of cells; 2 = moderate staining involving more than 70% of cells; and 3 = strong staining involving more than 70% of cells.

### Statistics.

All data are presented as the mean ± standard error of the mean (SEM), and sample sizes are indicated in the graphs or figure legends. All studies were conducted with a minimum of 3 technical and biological replicates. Statistical significance was set at *P* less than 0.05. The statistical analyses, including 2-tailed Student’s unpaired *t* test, 1-way ANOVA with Tukey’s multiple-comparison test, 2-way ANOVA with Tukey’s or Holm-Šidák multiple-comparison test, χ^2^ tests, and Fisher’s exact tests, were performed using GraphPad Prism version 10.4.0 for Windows. *P* < 0.05 was considered significant. If the graphs do not display statistical annotations (asterisks) indicating significance, the results are not statistically significant (*P* > 0.05). Statistical analyses were confirmed with the Cancer Biostatistics Shared Resource at Herbert Irving Comprehensive Cancer Center.

### Study approval.

All animal work and studies involving patient tissues were approved by the IRB or IACUC of Columbia University (see IRB protocol numbers above). Written informed consent was obtained from all participants prior to their involvement in the study, in accordance with IRB guidelines.

### Data availability.

Values for all data points in graphs are reported in the Supporting Data Values file. RNA-Seq data were deposited in the NCBI’s dbGaP database under the accession number phs003965.v1.p1. Mouse models, 3D organoid lines, and engineered 2D cell lines are available from the corresponding author under a material transfer agreement with Columbia University.

## Author contributions

AES and AKR conceptualized the study. Data curation and methodology were performed by AES and SPF. Formal analysis was conducted by AES, SPF, and AJKS. Funding was acquired by AES, CJL, PAS, and AKR. Investigations were carried out by AES, KS, SWK, and NN. Project administration and supervision were managed by AES and AKR. Resources were provided by AES, DAC, JTG, and DD. Validation was performed by AES and SPF. Visualization was done by AES. The original draft was written by AES, SWK, and AKR. NV, CJL, PAS, and AKR contributed to review and editing.

## Supplementary Material

Supplemental data

Unedited blot and gel images

Supporting data values

## Figures and Tables

**Table 2:**
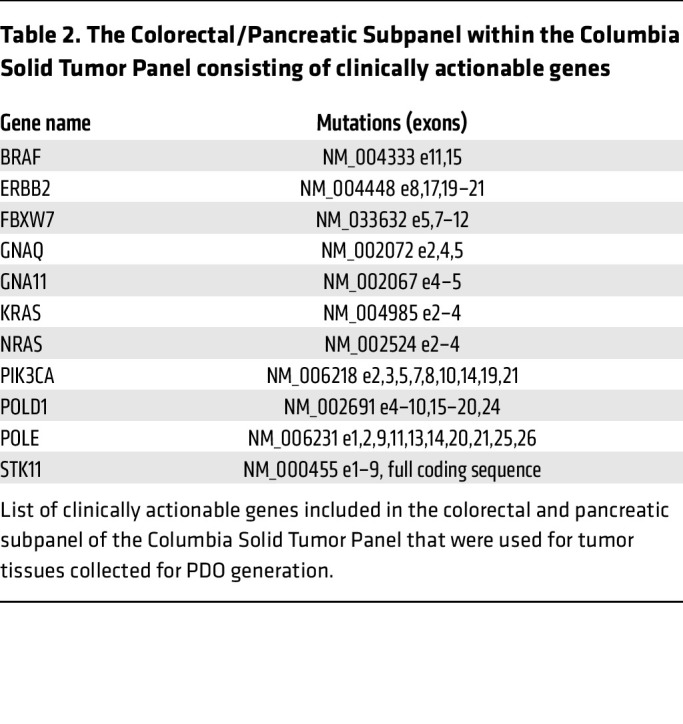
The Colorectal/Pancreatic Subpanel within the Columbia Solid Tumor Panel consisting of clinically actionable genes

**Table 1:**
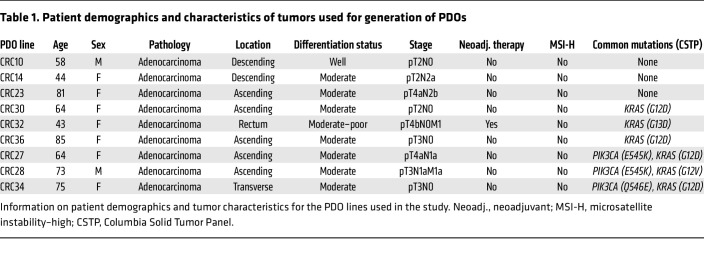
Patient demographics and characteristics of tumors used for generation of PDOs

## Version 1 01/14/2025

In-Press Preview

## Version 2 04/15/2025

Electronic publication

## References

[1] Siegel RL (2024). Cancer statistics, 2024. CA Cancer J Clin.

[2] https://www.cancer.org/cancer/types/colon-rectal-cancer/detection-diagnosis-staging/survival-rates.html.

[3] Vogelstein B (1988). Genetic alterations during colorectal-tumor development. N Engl J Med.

[4] Madison BB (2013). LIN28B promotes growth and tumorigenesis of the intestinal epithelium via Let-7. Genes Dev.

[5] Sugiura K (2023). LIN28B promotes cell invasion and colorectal cancer metastasis via CLDN1 and NOTCH3. JCI Insight.

[6] Polesskaya A (2007). Lin-28 binds IGF-2 mRNA and participates in skeletal myogenesis by increasing translation efficiency. Genes Dev.

[7] Xu B, Huang Y (2009). Histone H2a mRNA interacts with Lin28 and contains a Lin28-dependent posttranscriptional regulatory element. Nucleic Acids Res.

[8] Qiu C (2010). Lin28-mediated post-transcriptional regulation of Oct4 expression in human embryonic stem cells. Nucleic Acids Res.

[9] Wilbert ML (2012). LIN28 binds messenger RNAs at GGAGA motifs and regulates splicing factor abundance. Mol Cell.

[10] Hafner M (2013). Identification of mRNAs bound and regulated by human LIN28 proteins and molecular requirements for RNA recognition. RNA.

[11] Tu HC (2015). LIN28 cooperates with WNT signaling to drive invasive intestinal and colorectal adenocarcinoma in mice and humans. Genes Dev.

[12] King CE (2011). LIN28B promotes colon cancer progression and metastasis. Cancer Res.

[13] Hamano R (2012). High expression of Lin28 is associated with tumour aggressiveness and poor prognosis of patients in oesophagus cancer. Br J Cancer.

[14] Suzuki K (2021). LIN28B induces a differentiation program through CDX2 in colon cancer. JCI Insight.

[15] Fruman DA (2017). The PI3K pathway in human disease. Cell.

[16] Vanhaesebroeck B (2010). PI3K: from the bench to the clinic and back. Curr Top Microbiol Immunol.

[17] Weng QP (1995). Phosphatidylinositol 3-kinase signals activation of p70 S6 kinase in situ through site-specific p70 phosphorylation. Proc Natl Acad Sci U S A.

[18] Burgering BM, Coffer PJ (1995). Protein kinase B (c-Akt) in phosphatidylinositol-3-OH kinase signal transduction. Nature.

[19] Vanhaesebroeck B (2021). PI3K inhibitors are finally coming of age. Nat Rev Drug Discov.

[20] Markowitz SD, Bertagnolli MM (2009). Molecular origins of cancer: molecular basis of colorectal cancer. N Engl J Med.

[21] Okkenhaug K (2016). Targeting PI3K in cancer: impact on tumor cells, their protective stroma, angiogenesis, and immunotherapy. Cancer Discov.

[22] Liang S (2021). A PLCB1-PI3K-AKT signaling axis activates EMT to promote cholangiocarcinoma progression. Cancer Res.

[23] Maharati A, Moghbeli M (2023). PI3K/AKT signaling pathway as a critical regulator of epithelial-mesenchymal transition in colorectal tumor cells. Cell Commun Signal.

[24] Yaeger R (2018). Clinical sequencing defines the genomic landscape of metastatic colorectal cancer. Cancer Cell.

[25] Priestley P (2019). Pan-cancer whole-genome analyses of metastatic solid tumours. Nature.

[26] Mendelaar PAJ (2021). Whole genome sequencing of metastatic colorectal cancer reveals prior treatment effects and specific metastasis features. Nat Commun.

[27] Sartore-Bianchi A (2009). PIK3CA mutations in colorectal cancer are associated with clinical resistance to EGFR-targeted monoclonal antibodies. Cancer Res.

[28] Gowrikumar S (2021). A claudin-based molecular signature identifies high-risk, chemoresistant colorectal cancer patients. Cells.

[29] Malinowsky K (2014). Activation of the PI3K/AKT pathway correlates with prognosis in stage II colon cancer. Br J Cancer.

[30] Wang L (2014). PIK3CA mutations frequently coexist with EGFR/KRAS mutations in non-small cell lung cancer and suggest poor prognosis in EGFR/KRAS wildtype subgroup. PLoS One.

[31] Jhaveri K (2021). Phase I basket study of taselisib, an isoform-selective PI3K inhibitor, in patients with *PIK3CA*-mutant cancers. Clin Cancer Res.

[32] Juric D (2018). Phosphatidylinositol 3-kinase α-selective inhibition with alpelisib (BYL719) in PIK3CA-altered solid tumors: results from the first-in-human study. J Clin Oncol.

[33] Gouda MA, Subbiah V (2023). Precision oncology for BRAF-mutant cancers with BRAF and MEK inhibitors: from melanoma to tissue-agnostic therapy. ESMO Open.

[34] Varkaris A (2023). Discovery and clinical proof-of-concept of RLY-2608, a first-in-class mutant-selective allosteric PI3Kα inhibitor that decouples antitumor activity from hyperinsulinemia. Cancer Discov.

[35] Buckbinder L (2023). STX-478, a mutant-selective, allosteric PI3Kα inhibitor spares metabolic dysfunction and improves therapeutic response in PI3Kα-mutant xenografts. Cancer Discov.

[36] Shin AE (2023). Metastatic colorectal cancer: mechanisms and emerging therapeutics. Trends Pharmacol Sci.

[37] Madison BB (2002). Cis elements of the villin gene control expression in restricted domains of the vertical (crypt) and horizontal (duodenum, cecum) axes of the intestine. J Biol Chem.

[38] Srinivasan L (2009). PI3 kinase signals BCR-dependent mature B cell survival. Cell.

[39] Hoxhaj G, Manning BD (2020). The PI3K-AKT network at the interface of oncogenic signalling and cancer metabolism. Nat Rev Cancer.

[40] Moskaluk CA (2003). Cdx2 protein expression in normal and malignant human tissues: an immunohistochemical survey using tissue microarrays. Mod Pathol.

[41] el Marjou F (2004). Tissue-specific and inducible Cre-mediated recombination in the gut epithelium. Genesis.

[42] Guda K (2003). Multistage gene expression profiling in a differentially susceptible mouse colon cancer model. Cancer Lett.

[43] Li C (2022). Mouse models for application in colorectal cancer: understanding the pathogenesis and relevance to the human condition. Biomedicines.

[44] Shin AE (2023). F4/80^+^Ly6C^high^ macrophages lead to cell plasticity and cancer initiation in colitis. Gastroenterology.

[45] Andre F (2019). Alpelisib for *PIK3CA*-mutated, hormone receptor-positive advanced breast cancer. N Engl J Med.

[46] Razavi P (2020). Alterations in *PTEN* and *ESR1* promote clinical resistance to alpelisib plus aromatase inhibitors. Nat Cancer.

[47] Kim SK (2014). A nineteen gene-based risk score classifier predicts prognosis of colorectal cancer patients. Mol Oncol.

[48] Manning BD, Toker A (2017). AKT/PKB signaling: navigating the network. Cell.

[49] Saxton RA, Sabatini DM (2017). mTOR signaling in growth, metabolism, and disease. Cell.

[50] Turner NC (2023). Capivasertib in hormone receptor-positive advanced breast cancer. N Engl J Med.

[51] Taketo MM, Edelmann W (2009). Mouse models of colon cancer. Gastroenterology.

[52] Hung KE (2010). Development of a mouse model for sporadic and metastatic colon tumors and its use in assessing drug treatment. Proc Natl Acad Sci U S A.

[53] Boutin AT (2017). Oncogenic *Kras* drives invasion and maintains metastases in colorectal cancer. Genes Dev.

[54] Varga J (2020). AKT-dependent NOTCH3 activation drives tumor progression in a model of mesenchymal colorectal cancer. J Exp Med.

[55] Schwitalla S (2013). Loss of p53 in enterocytes generates an inflammatory microenvironment enabling invasion and lymph node metastasis of carcinogen-induced colorectal tumors. Cancer Cell.

[56] O’Rourke KP (2017). Transplantation of engineered organoids enables rapid generation of metastatic mouse models of colorectal cancer. Nat Biotechnol.

[57] de Sousa e Melo F (2017). A distinct role for Lgr5^+^ stem cells in primary and metastatic colon cancer. Nature.

[58] Yuan W (2013). Conditional activation of Pik3ca(H1047R) in a knock-in mouse model promotes mammary tumorigenesis and emergence of mutations. Oncogene.

[59] Vasan N, Cantley LC (2022). At a crossroads: how to translate the roles of PI3K in oncogenic and metabolic signalling into improvements in cancer therapy. Nat Rev Clin Oncol.

[60] Mitchell CB, Phillips WA (2019). Mouse models for exploring the biological consequences and clinical significance of *PIK3CA* mutations. Biomolecules.

[61] Koren S, Bentires-Alj M (2013). Mouse models of PIK3CA mutations: one mutation initiates heterogeneous mammary tumors. FEBS J.

[62] Vlachogiannis G (2018). Patient-derived organoids model treatment response of metastatic gastrointestinal cancers. Science.

[63] Liu J (2020). Molecular dissection of CRC primary tumors and their matched liver metastases reveals critical role of immune microenvironment, EMT and angiogenesis in cancer metastasis. Sci Rep.

[64] Lee SE (2021). High concordance of genomic profiles between primary and metastatic colorectal cancer. Int J Mol Sci.

[65] Ham-Karim H (2023). Investigating genomic, proteomic, and post-transcriptional regulation profiles in colorectal cancer: a comparative study between primary tumors and associated metastases. Cancer Cell Int.

[66] Kamal Y (2019). Transcriptomic differences between primary colorectal adenocarcinomas and distant metastases reveal metastatic colorectal cancer subtypes. Cancer Res.

[67] Gray J (2010). Cancer: genomics of metastasis. Nature.

[68] Janku F (2011). PIK3CA mutations frequently coexist with RAS and BRAF mutations in patients with advanced cancers. PLoS One.

[69] Voutsina A (2013). Combined analysis of KRAS and PIK3CA mutations, MET and PTEN expression in primary tumors and corresponding metastases in colorectal cancer. Mod Pathol.

[70] Luo Q (2020). KRAS and PIK3CA bi-mutations predict a poor prognosis in colorectal cancer patients: a single-site report. Transl Oncol.

[71] Serebriiskii IG (2019). Comprehensive characterization of RAS mutations in colon and rectal cancers in old and young patients. Nat Commun.

[72] Turner NC (2024). Inavolisib-based therapy in *PIK3CA*-mutated advanced breast cancer. N Engl J Med.

[73] Hollebecque A (2014). A phase Ib trial of LY2584702 tosylate, a p70 S6 inhibitor, in combination with erlotinib or everolimus in patients with solid tumours. Eur J Cancer.

[74] Tolcher A (2014). A phase I trial of LY2584702 tosylate, a p70 S6 kinase inhibitor, in patients with advanced solid tumours. Eur J Cancer.

[75] Larkin J (2014). Combined vemurafenib and cobimetinib in BRAF-mutated melanoma. N Engl J Med.

[76] Davies MA (2017). Dabrafenib plus trametinib in patients with BRAF^V600^-mutant melanoma brain metastases (COMBI-MB): a multicentre, multicohort, open-label, phase 2 trial. Lancet Oncol.

[77] Long GV (2017). Adjuvant dabrafenib plus trametinib in stage III BRAF-mutated melanoma. N Engl J Med.

[78] Sato T (2011). Long-term expansion of epithelial organoids from human colon, adenoma, adenocarcinoma, and Barrett’s epithelium. Gastroenterology.

