# Supplementary material for: LIN28B-mediated PI3K/AKT pathway activation promotes metastasis in colorectal cancer models
Source: J Clin Invest. 2025 Jan 14;135(8):e186035. doi: 10.1172/JCI186035 (PMC11996871; doi:10.1172/JCI186035)
Supplement: Supplemental data [file jci-135-186035-s249.pdf]

# SUPPLEMENTAL MATERIALS

## Materials and Methods

### Generation of LIN28B$^{high}$ cell lines

DLD-1 and LoVo cells were obtained from American Type Culture Collection (ATCC). To generate LoVo and DLD-1 cells with LIN28B expression, MSCV-PIG-LIN28B and empty vector control plasmids, generously provided by Joshua Mendell (Department of Molecular Biology, University of Texas Southwestern Medical Center, Dallas, Texas, USA), were used for transfection. Phoenix A cells were transfected with 30 μg of plasmid DNA. Transfection efficiency was assessed by monitoring GFP expression via light microscopy prior to virus collection. Viral supernatants were collected 48 hours post-transfection, filtered through a 0.45 μm membrane, flash-frozen in liquid nitrogen, and stored at -80°C. LoVo and DLD-1 cells were then infected by adding the virus-containing media supplemented with 4 μg/mL polybrene, followed by centrifugation at 1,000 × g for 90 minutes. Following infection, cells were selected with 2 μg/mL puromycin, expanded, and sorted for high GFP intensity to ensure corresponding high LIN28B expression. The expanded cells were maintained in DMEM (Corning 10-013-CV) supplemented with 10% fetal bovine serum (FBS; Cytiva SH30071) and 1% penicillin-streptomycin (Sigma-Aldrich P4333).

### Mouse colonic organoid culture

Mice were euthanized using $CO_2$, and colons were isolated, flushed with cold PBS, and opened longitudinally. The tissue was cut into 5 mm pieces from the distal to mid colon and transferred to 50 ml conical tubes containing 10 ml of cold PBS. The tissue in PBS was vigorously shaken 10

times, after which the PBS was discarded and replaced with fresh PBS; this washing process was repeated three times. Tissue pieces were then incubated in 25 ml of PBS supplemented with 20 mM EDTA on a rotator at 4°C. After removing the supernatant, tissues were resuspended in 9 ml of cold PBS and 1 ml of FBS, pipetted 12 times using a 10 ml serological pipette, and passed through a 100 µm cell strainer. The strained mixture was centrifuged at 300 x g for 5 minutes at 4°C. The supernatant was discarded, and the pellet was washed with cold Advanced DMEM/F-12 (Fisher Scientific 12-634-028). The pellet was then resuspended in growth factor-reduced Matrigel (Corning 356231) at a density of 100-200 crypts per 50 µL and plated in 50 µL aliquots on a pre-warmed 24-well plate (Corning 3526). After incubation at 37°C for 5-10 minutes to allow the Matrigel to solidify, 500 µL of warm "Organoid Growth Media" containing DMEM/F-12 supplemented with 10 mM HEPES (Gibco 15630), GlutaMAX (Gibco 35050), penicillin-streptomycin, B-27 supplement (Gibco 17504), N-2 supplement (Gibco 17502), and 1 mM N-acetylcysteine (Selleck Chemicals S1623) was added. Necessary growth factors specific to colon organoids were also added to the media: 50 ng/mL recombinant murine EGF (PeproTech 315-09), 100 ng/mL recombinant murine Noggin (PeproTech 250-38), 2% R-spondin 3-Fc fusion protein conditioned medium (ImmunoPrecise Antibodies, R001), and 0.5 nM WNT surrogate-Fc fusion protein (ImmunoPrecise Antibodies N001). Media were refreshed every 2-3 days, and organoids were passaged every 1-2 weeks as needed.

**Mouse colonic tumor organoid culture**

Colonic tumors were collected from mice, and the underlying muscle layers and fatty tissues were removed using scalpel/scissors. The remaining tumor tissue was chopped into 1-2 mm fragments and washed three times in cold dPBS containing 0.1 mg/mL Primocin (InvivoGen ant-pm). The

tumor fragments were then digested in 2 mL of “Organoid Base Media” (see above) with 0.1 mg/mL Primocin (InvivoGen ant-pm) and Amphotericin B (Sigma A2942), along with Enzymes H, R, and A from the Tumor Dissociation Kit (Miltenyi 130-095-929) and 500U DNase I (Roche 4716728001). The digestion process was carried out for 30 minutes at 37°C on a rotator. Post-digestion, the supernatant was strained through a 100 μm cell strainer to obtain a single-cell suspension. The cells were then washed with cold “Organoid Base Media” by centrifugation at 300G for 5 minutes at 4°C to remove any remaining digestion enzymes. The resulting cell pellet was resuspended to a concentration of 10,000-20,000 cells per 50 μL of Matrigel (Corning 356231) and plated as described above. Pre-warmed 500 μL of “Organoid Base Media” supplemented with 50 ng/mL recombinant murine EGF (PeproTech 315-09), 100 ng/mL recombinant murine Noggin (PeproTech 250-38), Amphotericin B and Antibiotic-Antimycotic (Gibco 15240) were added to each well. Amphotericin B and Antibiotic-Antimycotic were removed from the media after one week.

**Patient-derived organoid (PDO) culture**

Tumor tissues were obtained from patients undergoing elective surgery at NewYork-Presbyterian/Columbia University Irving Medical Center with written informed consent under the protocol approved by the University of Columbia Institutional Review Board (IRB; protocol number AAAT8778). To isolate cells for organoid cultures, CRC tumors were collected from surgical specimens, and the underlying muscle layers and fatty tissues were removed using scissors. The remaining tumor tissue was chopped into 1-2 mm fragments and washed three times in cold dPBS containing 0.1 mg/mL Primocin (InvivoGen ant-pm). The tissue fragments were then digested in 5 mL of “Organoid Base Media” (see above) with 0.1 mg/mL Primocin (InvivoGen

ant-pm) and Amphotericin B (Sigma A2942), along with Enzymes H, R, and A from the Tumor Dissociation Kit (Miltenyi 130-095-929) and 500U DNase I (Roche 4716728001). The digestion process was carried out for 45 minutes at 37°C on a rotator. Post-digestion, the supernatant was strained through a 100 μm cell strainer to obtain a single-cell suspension. The cells were then washed with cold "Organoid Base Media" by centrifugation at 300G for 5 minutes at 4°C. The resulting cell pellet was resuspended to a concentration of 10,000-20,000 cells per 50 μL of Matrigel (Corning 356237). 50 μL of the cell-Matrigel mixture was plated into each well of a 24-well plate (Corning 3526) and incubated for 5-10 minutes at 37°C to solidify the Matrigel. After incubation, 500 μL of "Organoid Base Media" supplemented with 50 ng/mL h-EGF (Peprotech, AF-100-15), 50 ng/mL h-FGF2 (Peprotech, 100-18B), 100 ng/mL h-IGF1 (PeproTech, 100-11), 0.5 μM A83-01 (Tocris, 2939), 10 μM Y-27632 (SelleckChem, S1049), and 1% Noggin conditioned media (ImmunoPrecise Antibodies, N002), pre-warmed to 37°C, was added to each well. Amphotericin B and Antibiotic-Antimycotic were removed from the media after one week. To note, the protocol outlined above is also effective for tumor tissues that have been frozen in liquid nitrogen for up to 6 months.

**Quantification of organoid growth**

Organoid growth was monitored using the BioTek BioSpa Live Cell Analysis System and imaged with BioTek Gen5 Software for Imaging & Microscopy. Organoids were grown in 24-well plates, with 12 images captured per well at 4x magnification. Each image included a z-stack of 10 levels to ensure full coverage of the organoids within the Matrigel. The z-stack images were compressed into a single plane (z-projection) and subsequently stitched to form a complete image of each well. Images were acquired every 24 hours to track organoid growth over time. The stitched images

were analyzed using the Analyze Particles function in ImageJ FIJI software. The following parameters were applied: particle size range set to 50-Infinity (pixel$^2$) and circularity between 0.30-1.00. Holes within organoids were included, and organoids touching the edges were excluded from the analysis. The average size of organoids for each well were normalized to the average organoid size on day 0 (the first day of imaging, prior to treatments) for each subsequent day.

**Portal vein injection of LIN28B$^{high}$ CRC cells**

All animal studies were approved by the Institutional Animal Care and Use Committee (IACUC) at Columbia University, and all experiments were conducted in compliance with the NIH guidelines for animal research. Five weeks old male athymic nude mice (CrTac NCr-Foxn1$^{nu}$) were obtained from Taconic Biosciences (model NCRNU). After a week of acclimation, 6-week-old mice were anesthetized with isoflurane. Each mouse received an injection of $2.0 \times 10^6$ CRC cells suspended in 100 μL of PBS directly into the portal vein via open laparotomy using a 31G needle. Six weeks post-injection, the mice were euthanized by $CO_2$ inhalation. After excising the livers, GFP+ cells, indicative of liver metastases, were visualized using a Keyence fluorescence microscope. The livers were then fixed in 10% neutral buffered formalin for 24 hours, transferred to 70% ethanol, and subsequently embedded in paraffin. 5 μm liver sections were collected at every 300 μm and were prepared and stained with hematoxylin and eosin (H&E). The areas of liver metastases were quantified by tracing each tumor using ImageJ software.

**Glucose tolerance test (GTT)**

GTT was conducted on 12-week-old *Vil*$^{Cre}$*;R26*$^{WT/WT}$ and *Vil*$^{Cre}$*;R26*$^{Pik3ca/Pik3ca}$ mice. The mice were fasted for 16 hours the night before the test. On the day of the test, the mice were weighed, and a small incision (1-2 mm) was made on the tail to collect a baseline blood glucose measurement using a calibrated glucometer. A glucose solution (Sigma G8769) was administered to the mice via gavage at a dose of 2 g/kg of body weight. Following glucose administration, blood glucose levels were measured at designated time points: 15, 30, 60, 90, and 120 minutes post-gavage.

**Protein collection from 2D cell lines**

After washing with PBS, LoVo and DLD-1 cells were harvested and lysed using Cell Lysis Buffer (Cell Signaling Technology 9803) supplemented with 1 mM phenylmethylsulfonyl fluoride (PMSF; Cell Signaling Technology 8553S) and a protease and phosphatase inhibitor cocktail (Thermo Scientific, 78443). Cells were vortexed three times at 5-minute intervals, and then centrifuged at 14,000 rpm for 15 minutes at 4°C. The supernatant containing proteins was collected for further analysis.

**Protein collection from 3D organoids**

Organoids were retrieved from Matrigel using cold Cell Recovery Solution (Corning 354253) and dissociated into single cells by incubation with pre-warmed 0.25% trypsin (Gibco 25200) on 850 RPM shaker for 5-7 minutes. After dissociation, trypsin was neutralized using a 1:1 dilution of trypsin inhibitor (Sigma-Aldrich T9128), and cell pellets were collected by centrifugation. Cells were lysed by resuspension in Cell Lysis Buffer (Cell Signaling Technology 9803) supplemented with 1 mM phenylmethylsulfonyl fluoride (PMSF; Cell Signaling Technology 8553S) and a

protease/phosphatase inhibitor cocktail (Thermo Scientific 78443). The lysates were incubated on ice for 30 minutes with intermittent vortexing every 10 minutes. Following incubation, the samples were centrifuged at 14,000 rpm for 15 minutes at 4°C. The supernatant containing the proteins was collected for further analysis.

**Western blots**

Protein concentrations were determined using the Bradford method with Protein Assay Dye Reagent Concentrate (Bio-Rad 5000006). A total of 25 µg of protein from each sample was loaded onto Mini-PROTEAN TGX Precast Protein Gels, 4-15% (Bio-Rad 4561086) and separated using Tris-Glycine-SDS Running Buffer (Boston BioProducts BP-150). Proteins were transferred to a Trans-Blot Turbo Nitrocellulose Transfer Packs, Midi, 0.2 µm (Bio-Rad 1704159). Immunoblotting was conducted with primary antibodies (Supplemental Table 1) and visualized using IRDye secondary antibodies (LI-COR Biosciences 926-68070, 926-32211). Measured protein levels were normalized to either GAPDH or β-actin as an endogenous control.

**Immunohistochemistry (IHC)**

5 µm sections were warmed in a 60°C oven for 15 minutes. The tissues were deparaffinized, rehydrated, and given a final rinse in deionized water (di$H_2O$). Antigen retrieval was performed using a pressure cooker with either a pH 6 buffer (EMS 62706-10) or a pH 9 buffer (Abcam ab93684), depending on the primary antibody. Following antigen retrieval, endogenous peroxidase activity was quenched with 3% hydrogen peroxide for 15 minutes. The tissues were then blocked sequentially with Avidin D blocking reagent (Sigma A9275), Biotin blocking reagent (Sigma

B4501), and StartingBlock Blocking Buffer (ThermoFisher 37539), each for 15 minutes. Primary antibodies were diluted in a solution of 1x PBS with 0.1% BSA and 0.2% Triton X, and applied to the tissues in a humid chamber overnight at 4°C. On the following day, the slides were washed and incubated with SignalStain Boost IHC Detection reagent (Cell Signaling 8114S), which includes a rabbit secondary antibody, for 30 minutes. The signal was developed using a DAB Substrate Kit for Peroxidases (Vector SK4100) under microscopic observation to determine the optimal development time for each antibody. Counterstaining was performed with Harris hematoxylin (Leica 3801560) for 3 seconds, followed by washing and dehydration. The slides were mounted with Cytoseal, cover slipped, and allowed to dry before analysis.

**Wound healing (scratch) assay**

Confluent monolayer cells in 24-well plates were scratched using a 200 μL pipette tip to create a uniform wound. Images of wound closure were captured every 6 or 12 hours using BioTek BioSpa Live Cell Analysis System and BioTek Gen5 Software for Imaging & Microscopy. The wounded area was measured using ImageJ software as previously described (*1*). The wound-healing rate was normalized to the area of the initial scratch wound.

**QCM ECMatrix Cell Invasion Assay**

The ECM cell invasion assay was performed using the ECMatrix Cell Invasion Assay Kit (ECM550) as per the manufacturer's instructions. To prepare the inserts, 300 μL of warm DMEM was added to the interior of each insert and incubated at room temperature for 1 hour to rehydrate the ECM layer. Cells of interest were trypsinized to a concentration of 1 x $10^6$ cells/mL without

FBS. The media was removed from the inserts, and 500 µL of normal media (with or without alpelisib) was added to the lower chamber. Next, 300 µL of the cell suspension (without FBS) was added to each insert. The cells were incubated for 72 hours. For visualization of invaded cells, non-invading cells and the ECMatrix were gently removed from the inserts using a cotton swab. The inserts were then dipped into 500 µL of staining solution placed in the lower wells for 20 minutes. After staining, the inserts were washed several times in sterile water and air-dried. Invaded cells were counted under a microscope.

**Bulk RNA-sequencing**

STAR 2.7.10 was used to align trimmed fastq files to the reference genome (hg38), and the Picard CollectRnaSeqMetrics module (Picard) was used to evaluate alignments and perform initial quality control (QC). Gene expression was quantified with HTSeq, and these data were imported into RStudio (R 3.5) for DESeq2 (RRID: SCR_015687) analysis to determine differentially expressed genes across different conditions. For the analysis of publicly available RNA-seq datasets of primary colorectal tumors and matched liver metastases, data were retrieved from GSE50760 (*2*).

**Whole exome sequencing (WES)**

DNA was extracted from CRC PDO lines using the QIAamp DNA Micro Kit (56304) and the YY method according to the manufacturer's instructions. CRC cell lines were frozen on dry ice and shipped as frozen cell pellets to GENEWIZ from Azenta Life Sciences (at least $10^6$ cells/tube). Exon capture was performed by GENEWIZ using the Twist Biosciences Human Comprehensive Panel (South San Francisco, CA, USA). Library preparation was performed according to the

manufacturer's guidelines. Samples were sequenced (2 x 150 bp) to an estimated 30x coverage using the Illumina NovaSeqX platform. Whole exome sequencing data were analyzed using the Genome Analysis Toolkit pipeline (*3, 4*). Briefly, fastq files were analyzed using FastQC3 (v0.12.1) and adaptors were trimmed using bbduk (v38.90) (*5*). Trimmed reads were aligned to hg38 using bwa-mem2 (v2.2.1) (*6*) and sorted and indexed with samtools (v1.19) (*7*). Duplicates were marked with picard (v3.1.1) and base quality score recalibration was applied with GATK (v4.5.0.0). Variant calling was performed with Mutect2 (GATK) and calls were filtered using LearnReadOrientationModel, GetPileUpSummaries, CalculateContamination, and FilterMutectCalls (GATK). Filtered mutant calls were annotated using Funcotator (GATK). Data were visualized with Maftools (v2.8.5) (*8*).

**List of PI3K/AKT/mTOR pathway genes used for WES**

ACACA, ACTR2, ACTR3, ADCY2, AKT1, AKT1S1, AKT2, AKT3, AP2M1, ARF1, ARHGDIA, ARPC3, ATF1, CAB39, CAB39L, CALR, CAMK4, CDK1, CDK2, CDK4, CDKN1A, CDKN1B, CFL1, CLTC, CSNK2B, CXCR4, DAPP1, DDIT3, DEPDC5, DEPTOR, DUSP3, E2F1, ECSIT, EGFR, EIF4E, EIF4EBP1, FASLG, FGF17, FGF22, FGF6, GNA14, GNGT1, GRB2, GRK2, GSK3B, HRAS, HSP90B1, IL2RG, IL4, INPP4B, IRAK4, ITPR2, LCK, MAP2K3, MAP2K6, MAP3K7, MAPK1, MAPK10, MAPK8, MAPK9, MAPKAP1, MKNK1, MKNK2, MLST8, MTOR, MYD88, NCK1, NFKBIB, NGF, NOD1, NPRL2, NPRL3, PAK4, PDK1, PFN1, PIK3CA, PIK3CB, PIK3R1, PIK3R2, PIK3R3, PIKFYVE, PIN1, PITX2, PLA2G12A, PLCB1, PLCG1, PPP1CA, PPP2R1A, PPP2R1B, PRKAA2, PRKAG1, PRKAR2A, PRKCB, PTEN, PTPN11, RAC1, RAF1, RALB, RHEB, RICTOR, RIPK1, RIT1, RPS6, RPS6KA1, RPS6KA3, RPS6KB1, RPTOR, SFN, SLA, SLC2A1, SMAD2, SQSTM1, STAT2,

STK11, TBK1, THEM4, TIAM1, TNFRSF1A, TRAF2, TRIB3, TSC1, TSC2, UBE2D3, UBE2N, VAV3, YWHAB.

**Single cell RNA sequencing (scRNA-seq)**

Data from the Human Colon Cancer Atlas (c295) available on the Broad Institute's Single Cell Portal was utilized (https://singlecell.broadinstitute.org/single_cell). This dataset comprises 371,223 cells and provides a comprehensive single-cell transcriptomic landscape of human colon cancer (*9*).

## Supplemental Figures

## Supplemental Figure 1

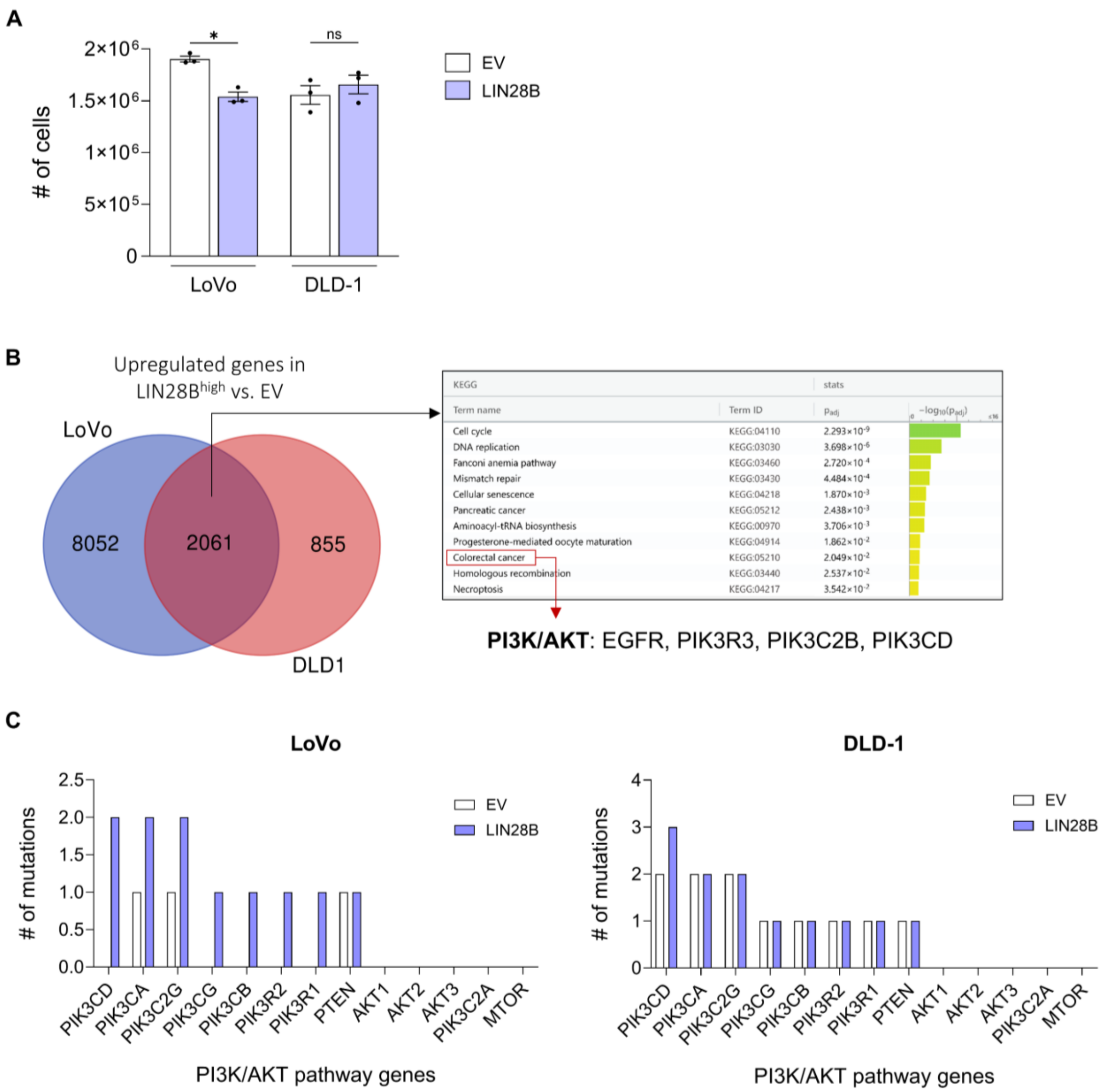


**Supplemental Figure 1. Bulk RNA-seq and WES of LIN28B$^{high}$ LoVo and DLD-1 cells. (A)** Empty vector (EV) control and LIN28B$^{high}$ CRC cells numbers were quantified after 48 hours of culture to assess differences in cell growth in vitro. **(B)** Venn diagram showing the overlap of upregulated genes in LIN28B$^{high}$ vs. EV control in LoVo and DLD-1 cells identified by bulk RNA-

seq. KEGG pathway analysis of the 2061 common upregulated genes highlights significant enrichment in the colorectal cancer pathway, including PI3K/AKT pathway genes (*EGFR*, *PIK3R3*, *PIK3C2B*, *PIK3CD*). **(C)** Number of mutations in PI3K/AKT pathway genes identified by WES in EV and LIN28B$^{high}$ LoVo (left) and DLD-1 (right) cells. Each bar represents the number of mutations for a specific gene in the pathway.

## Supplemental Figure 2

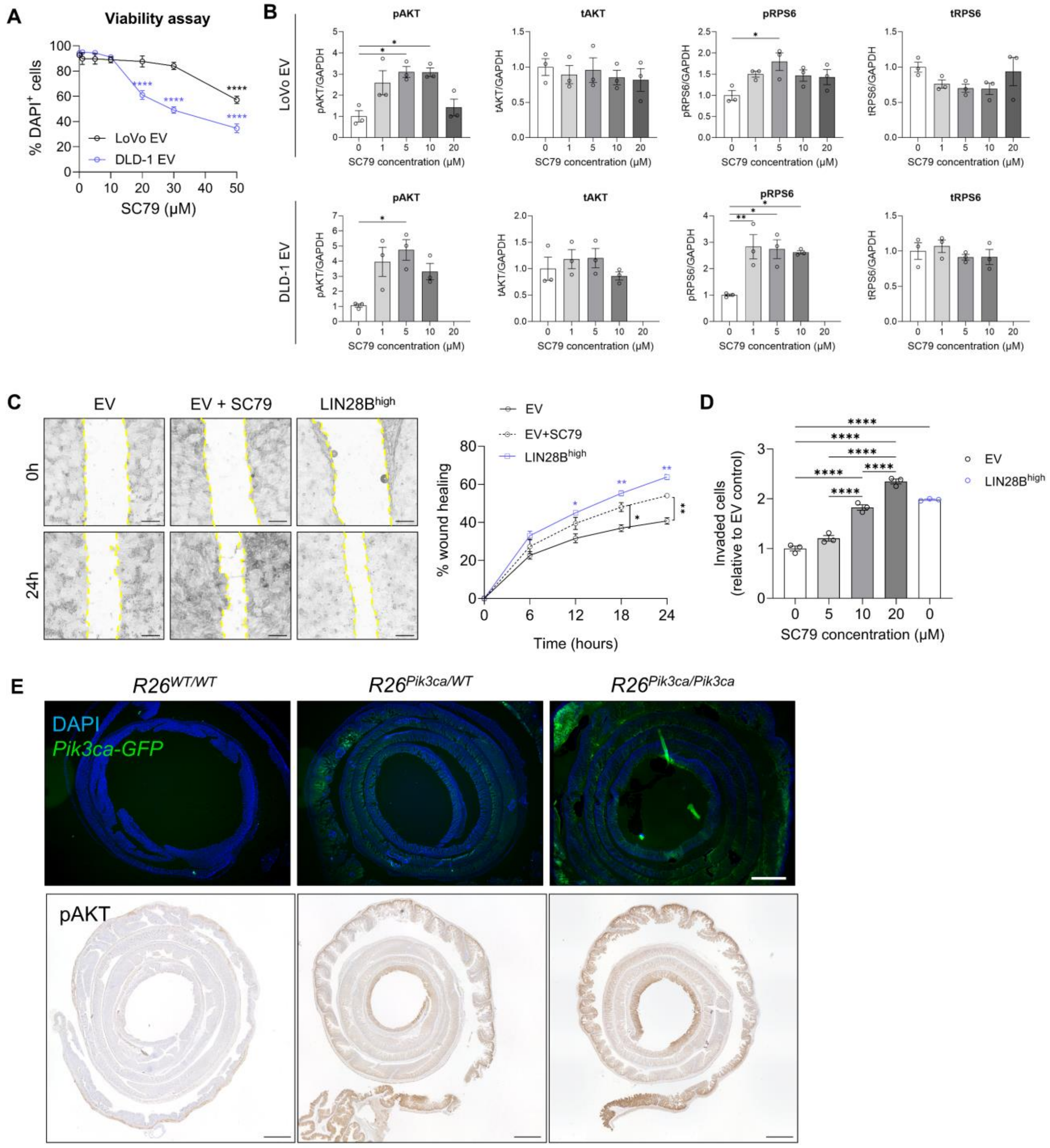


**Supplemental Figure 2. SC79-induced activation of the PI3K/AKT pathway enhances CRC cell migration and invasion. (A)** Viability assay of LoVo and DLD-1 cells treated with increasing concentrations of SC79, a pan-AKT activator. The percentage of DAPI+ cells was measured by

flow cytometry and compared within each cell line at each concentration against the control (0 µM) (n=3; two-way ANOVA, mean ± SEM). **(B)** Immunoblot analysis of pAKT (Ser473), tAKT, pRPS6 (Ser235/236), and tRPS6 in LoVo and DLD-1 cells expressing EV treated with 0, 5, 10, or 20 µM SC79. Quantification relative to GAPDH is shown (n=3; one-way ANOVA, mean ± SEM). **(C)** Wound healing assay showing cell migration of LoVo cells expressing EV, EV + 5 µM SC79, or LIN28B$^{high}$, measured every 6 hours for 24 hours. Representative images of wound closure are shown on the left. Quantification of wound healing percentage over time is shown on the right (n=3; two-way ANOVA, mean ± SEM). **(D)** Transwell ECM invasion assay of LoVo EV cells treated with 0, 5, 10, or 20 µM SC79. Quantification of invaded cells relative to the control (0 µM SC79) is shown (n=3; one-way ANOVA, mean ± SEM). **(E)** Representative images of GFP fluorescence and pAKT (Ser473) in colonic tissues from $R26^{WT/WT}$, $R26^{Pik3ca/WT}$, and $R26^{Pik3ca/Pik3ca}$ mice. Scale bars = 1 mm. *P<0.05, **P<0.01, ***P<0.001, ****P<0.0001.

**Supplemental Figure 3**

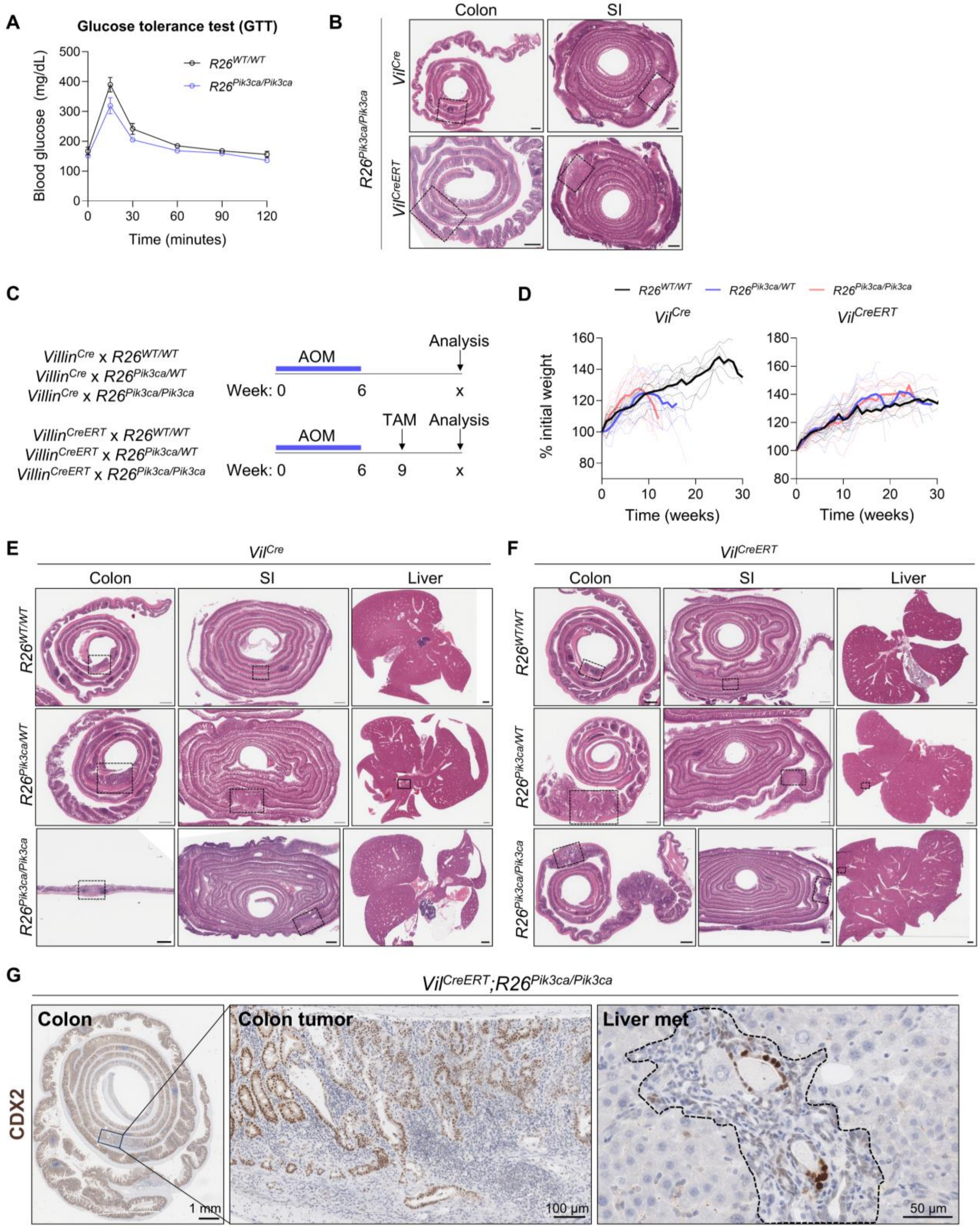

**Supplemental Figure 3. *Villin*$^{Cre}$ and *Villin*$^{CreERT}$ mice crossed to *R26*$^{Pik3ca}$ show spontaneous tumorigenesis, tumor invasiveness, and liver metastasis formation. (A)** Glucose tolerance test (GTT) of *Villin*$^{Cre}$*;R26*$^{WT/WT}$ and *Villin*$^{Cre}$*;R26*$^{Pik3ca/Pik3ca}$ mice. Blood glucose levels were measured at the indicated time points after glucose administration (mean ± SEM; n=5). **(B)** Representative H&E images of the colon, SI, and liver from *Villin*$^{Cre}$ and *Villin*$^{CreERT}$ mice crossed to *R26*$^{Pik3ca/Pik3ca}$ mice. Scale bars = 1000 μm. **(C)** Experimental setup for AOM-induced CRC model. **(D)** Quantification of changes in body weight of *Villin*$^{Cre}$ and *Villin*$^{CreERT}$ mice crossed to *R26*$^{WT/WT}$, *R26*$^{Pik3ca/WT}$, and *R26*$^{Pik3ca/Pik3ca}$ mice treated with AOM. Thick solid lines represent the average weight of all mice in the group, while thin dotted lines represent individual mice. **(E and F)** Representative H&E images of the colon, SI, and liver from *Vil*$^{Cre}$ (E) and *Vil*$^{CreERT}$ (F) mice crossed to *R26*$^{WT/WT}$, *R26*$^{Pik3ca/WT}$, and *R26*$^{Pik3ca/Pik3ca}$ mice. Scale bars = 1000 μm. **(G)** Representative IHC images of CDX2 staining in the colon, colon tumor, and liver metastasis from *Vil*$^{CreERT}$*;R26*$^{Pik3ca/Pik3ca}$ mice. Scale bars = 1 mm (colon), 100 μm (colon tumor), and 50 μm (liver metastasis).

**Supplemental Figure 4**

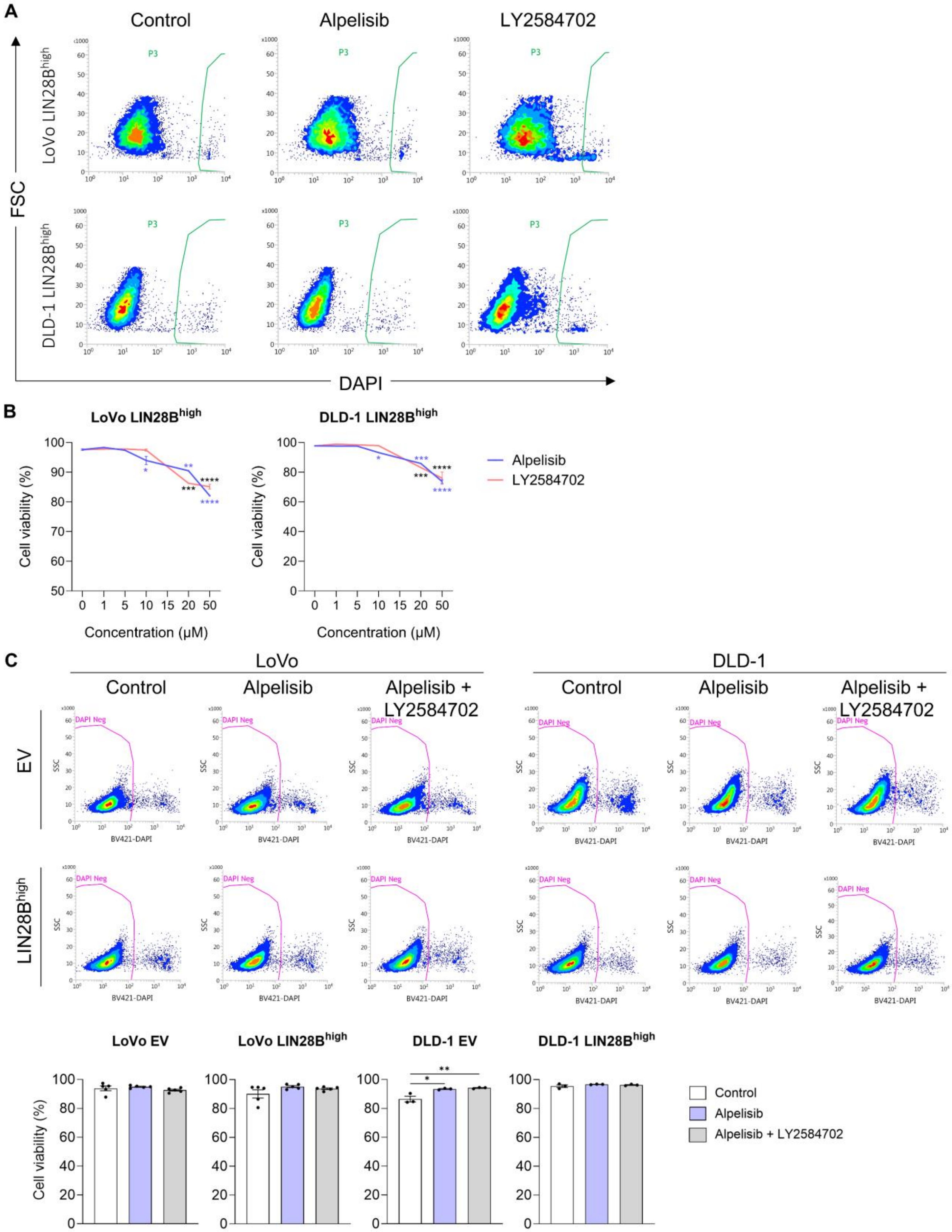

**Supplemental Figure 4. Viability assay for alpelisib and LY2584702 on CRC cell lines. (A)** Representative flow cytometry analysis showing the viability of LoVo LIN28B$^{high}$ and DLD-1 LIN28B$^{high}$ cells treated with control (vehicle), alpelisib (50 μM), or LY2584702 (50 μM) for 48 hours. Cells were stained with DAPI to assess cell viability. FSC = forward scatter. **(B)** Quantification of cell viability of LoVo LIN28B$^{high}$ and DLD-1 LIN28B$^{high}$ cells treated with increasing concentrations of alpelisib or LY2584702 for 48 hours. Viability was measured by DAPI staining and flow cytometry. Data are presented as the percentage of viable cells relative to the vehicle-treated control (mean ± SEM; n=2; two-way ANOVA). **(C)** Representative flow cytometry plots showing the viability of LoVo and DLD-1 EV and LIN28B$^{high}$ cells after treatment with control (vehicle), alpelisib (5 μM), or the combination of alpelisib (5 μM) and LY2584702 (5 μM) for 24 hours. Cells were stained with DAPI to quantify live cells (mean ± SEM, one-way ANOVA).*P<0.05, **P<0.01, ***P<0.001, ****P<0.0001.

**Supplemental Figure 5**

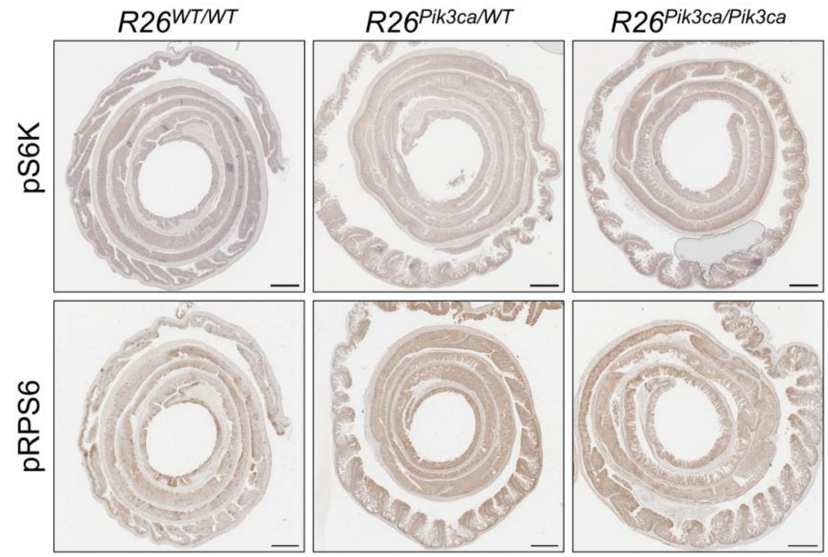


**Supplemental Figure 5. IHC staining of pS6K and pRPS6 in *Villin*$^{Cre}$;*R26*$^{Pik3ca}$ mice.** Representative IHC images of pS6K and pRPS6 in colonic tissues from *R26*$^{WT/WT}$, *R26*$^{Pik3ca/WT}$, and *R26*$^{Pik3ca/Pik3ca}$ mice crossed with *Villin*$^{Cre}$. Scale bars = 1000 μm.

# Supplemental Figure 6

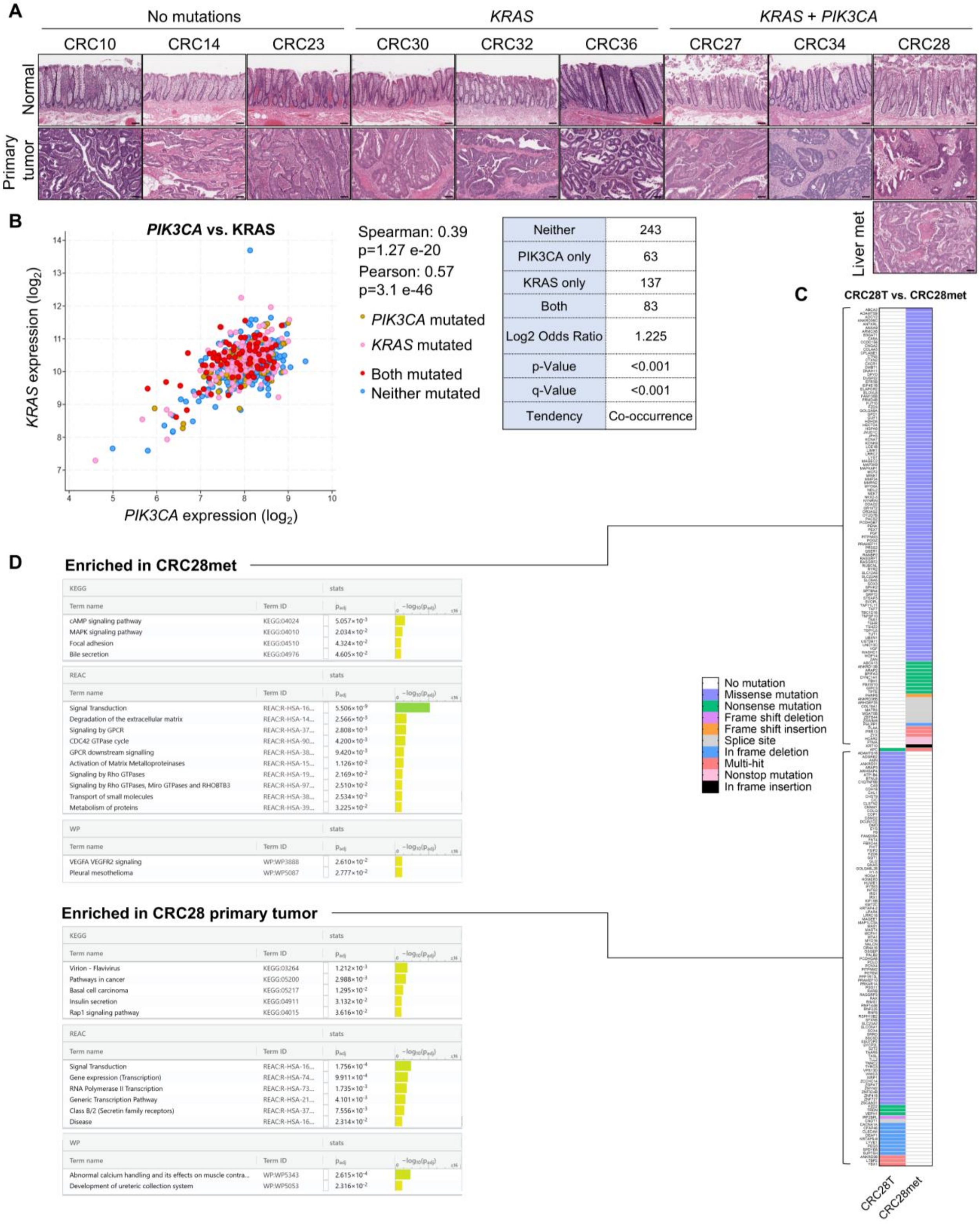

**Supplemental Figure 6. Characterization of PDOs. (A)** Representative H&E images of normal colon tissue and primary tumors from CRC23, CRC32, CRC27, and CRC28 patients. Additional image of liver metastasis for CRC28. These tissues were used to PDOs. Scale bars = 100 μm. **(B)** Scatter plot showing the correlation between *PIK3CA* and *KRAS* expression (log2) in CRC samples from TCGA. Samples are color-coded based on mutation status: *PIK3CA* mutated, *KRAS* mutated, both mutated, or neither mutated. Statistical analysis includes Spearman and Pearson correlation coefficients. Table summarizes the co-occurrence analysis of *PIK3CA* and *KRAS* mutations. **(C)** Heatmap comparing the mutation profiles of CRC28 primary colonic tumor (CRC28T) versus liver metastasis (CRC28met) PDO lines, identified by WES. Only genes with mutations are shown, with mutation types color-coded as indicated in the legend. **(D)** KEGG, Reactome, and WikiPathways enrichment analysis for genes mutated in CRC28met but not in CRC28T, and vice versa. The top pathways enriched in each comparison are shown.

**Supplemental Figure 7**

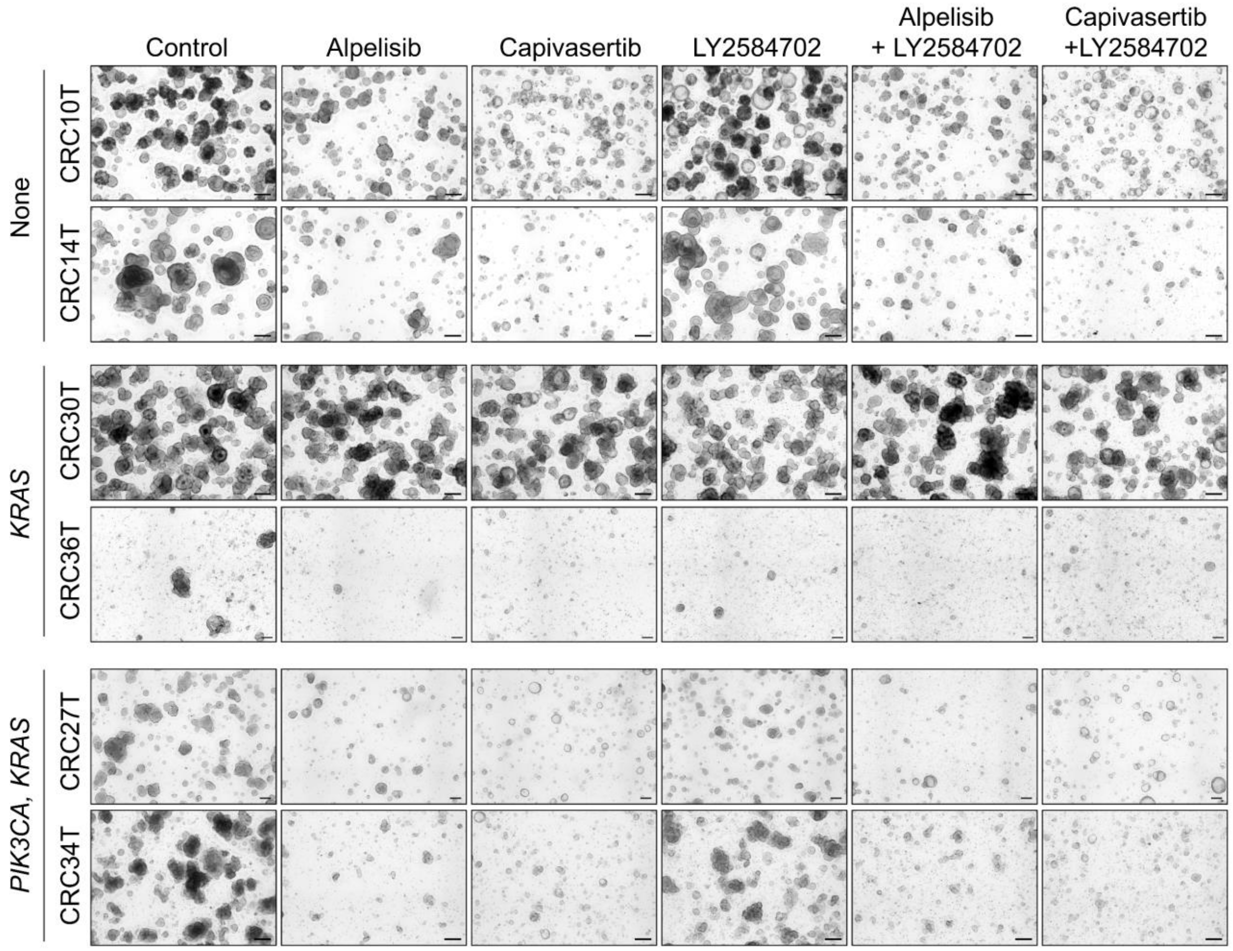


**Supplemental Figure 7. Representative images of PDOs treated with PI3K/AKT/MTORC1 pathway inhibitors.** Representative bright-field images of PDOs treated with control, alpelisib, capivasertib, LY2584702, alpelisib + LY2584702, or capivasertib + LY2584702 every 3 days for 9 days. Scale bars = 100 μm.

**Supplemental Figure 8**

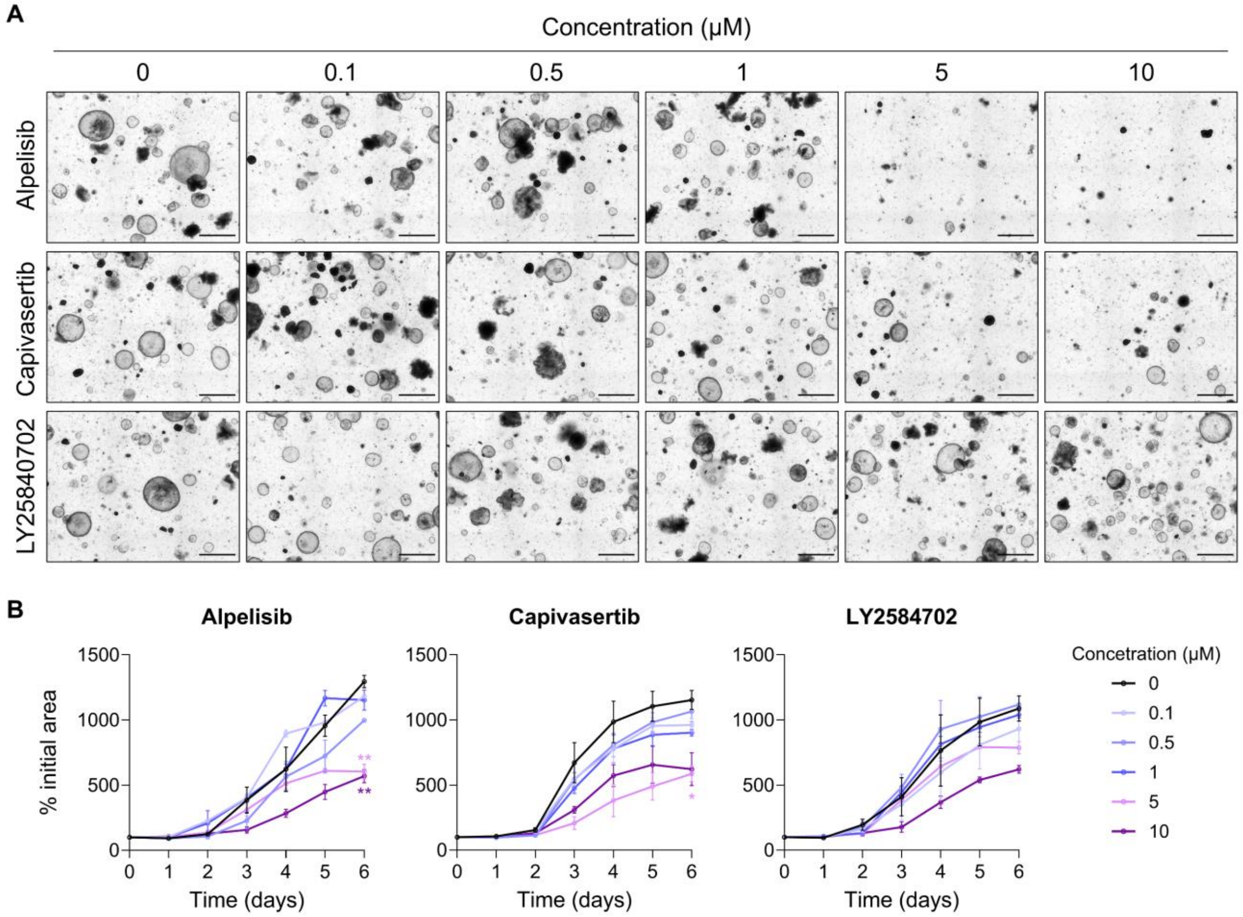


**Supplemental Figure 8. Dose-response analysis of alpelisib, capivasertib, and LY2584702 in *Vil$^{Cre}$;R26$^{Pik3ca/Pik3ca}$* mouse colonic tumor organoids. (A** and **B)** Representative bright-field images (A) and growth curves (B) of colonic tumor organoids derived from *Vil$^{Cre}$;R26$^{Pik3ca/Pik3ca}$* mice treated with control, alpelisib, capivasertib, or LY2584702 every 3 days for 6 days. Images are shown for day 6. Scale bars = 100 μm. Growth curve shows percent increase in initial area (n=3; two-way ANOVA, mean ± SEM).

**Supplemental Figure 9**

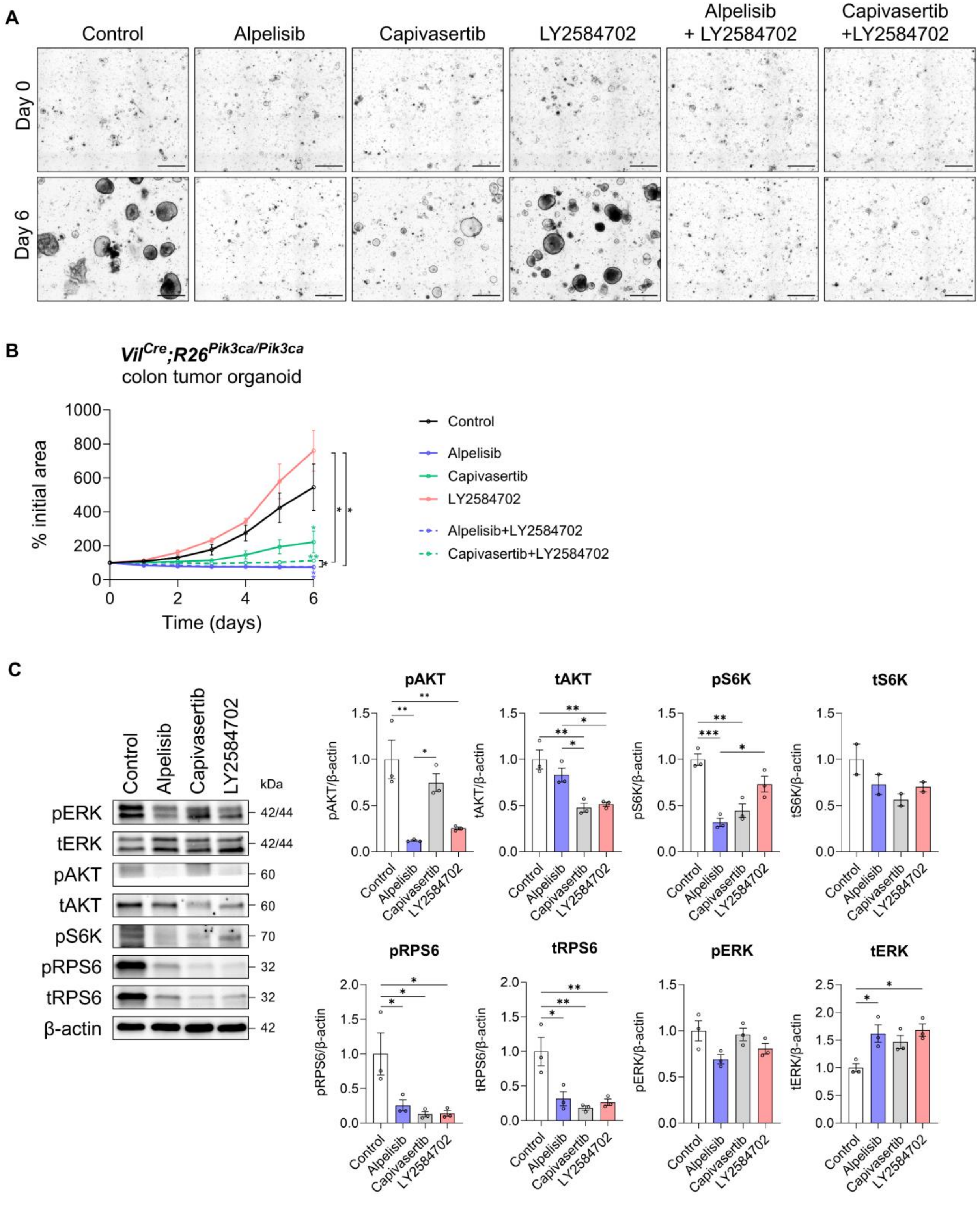

**Supplemental Figure 9. Organoids derived from colonic tumors of *Villin*$^{Cre}$*;R26*$^{Pik3ca/Pik3ca}$ mice are sensitive to PI3K pathway inhibitors. (A** and **B)** Representative bright-field images on day 0 and day 6 (A) and growth curves over a period of 6 days (B) of colonic tumor organoids treated with control, alpelisib, capivasertib, LY2584702, alpelisib + LY2584702, or capivasertib + LY2584702. Scale bars = 100 μm. (n=3; two-way ANOVA, mean ± SEM). **(C)** Representative Western blot images (left) and quantifications of protein levels normalized to β-actin (right) for PI3K/AKT pathway-related proteins in colonic tumor organoids treated with control, alpelisib, capivasertib, or LY2584702. (n=3; one-way ANOVA, mean ± SEM).

**Supplemental Figure 10**

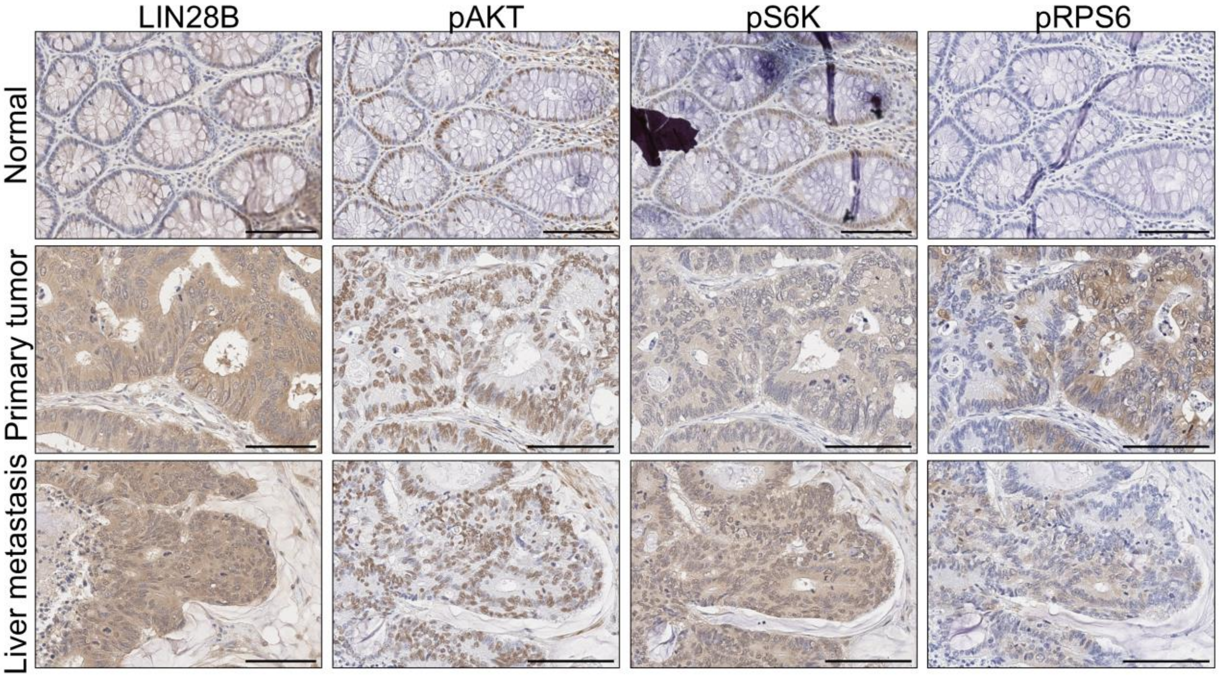


**Supplemental Figure 10. Serial sections of normal, primary tumor, and liver metastasis tissue stained for LIN28B and phosphorylated markers**. Representative IHC images of serial sections from "normal", "primary tumor", and "liver metastasis" tissues collected from patient #29, stained for LIN28B, pAKT, pS6K, and pRPS6. Scale bars = 100 µm.

**Supplemental Figure 11**

| # of cells | | | |
|---|---|---|---|
| Epithelial | Stage: T4 | | |
| 10027 | 3535 | cE01 (Stem/TA-like) | Normal |
| 4640 | 1260 | cE02 (Stem/TA-like/Immature Goblet) | Normal |
| 4894 | 1519 | cE03 (Stem/TA-like proliferating) | Normal |
| 7204 | 1965 | cE04 (Enterocyte 1) | Normal |
| 9967 | 2952 | cE05 (Enterocyte 2) | Normal |
| 5944 | 2014 | cE06 (Immature Goblet) | Normal |
| 1241 | 409 | cE07 (Goblet/Enterocyte) | Normal |
| 5339 | 2165 | cE08 (Goblet) | Normal |
| 6618 | 1389 | cE09 (Best4) | Normal |
| 1475 | 396 | cE10 (Tuft) | Normal |
| 151 | 65 | cE11 (Enteroendocrine) | Normal |
| 10028 | 20432 | cE01 (Stem/TA-like) | Tumor |
| 9186 | 4606 | cE02 (Stem/TA-like/Immature Goblet) | Tumor |
| 10028 | 16855 | cE03 (Stem/TA-like proliferating) | Tumor |
| 1903 | 759 | cE04 (Enterocyte 1) | Tumor |
| 3043 | 1457 | cE05 (Enterocyte 2) | Tumor |
| 4218 | 1914 | cE06 (Immature Goblet) | Tumor |
| 36 | 13 | cE07 (Goblet/Enterocyte) | Tumor |
| 745 | 168 | cE08 (Goblet) | Tumor |
| 251 | 69 | cE09 (Best4) | Tumor |
| 1374 | 478 | cE10 (Tuft) | Tumor |
| 399 | 114 | cE11 (Enteroendocrine) | Tumor |
| 1289 | 74013 | Undefined | |

**Supplemental Figure 11. Detailed legend and cell counts for each epithelial cell subtype depicted in Figure 8C.** A comprehensive legend detailing the annotation of epithelial cell subpopulations identified in the Human Colon Cancer Atlas single-cell RNA sequencing dataset (c295), shown in Figure 8C. The table lists cell subtype annotations (Normal or Tumor cells cE01-cE11), their functional designations, and corresponding cell counts for all epithelial cells and T4 stage tumor cells.

## Supplemental Tables

**Supplemental Table 1.** List of primary antibodies used for Western blot and IHC.

| Antibody | Catalog # | Application | Dilution |
|---|---|---|---|
| LIN28B | HPA061745 | Western blot | 1:1000 |
| | ab264334 | IHC | 1:1000 |
| Phospho-AKT (Ser473) | CST4051 | Western blot | 1:500 |
| | CST4060 | IHC | 1:50 |
| Total AKT | CST9272 | Western blot | 1:500 |
| Phospho-MTOR | CST5536 | Western blot | 1:1000 |
| Total MTOR | CST2983 | Western blot | 1:1000 |
| Phospho-S6K (Thr389/412) | ab2571 | Western blot | 1:500 |
| | | IHC | 1:100 |
| Total S6K | CST34475 | Western blot | 1:1000 |
| Phospho-RPS6 (Ser235/236) | CST2211 | Western blot | 1:1000 |
| | | IHC | 1:200 |
| Total RPS6 | CST2217 | Western blot | 1:1000 |
| Ki67 | ab16667 | IHC | 1:500 |
| GAPDH | ab8245 | Western blot | 1:2000 |
| β-actin | Sigma A5316 | Western blot | 1:10,000 |

## References


1. K. Sugiura*, Y. Masuike*, K. Suzuki*, A. E. Shin*, N. Sakai, H. Matsubara, M. Otsuka, P. A. Sims, C. J. Lengner, A. K. Rustgi, LIN28B promotes cell invasion and colorectal cancer metastasis via CLDN1 and NOTCH3. *JCI Insight* **8**, (2023). *co-first authors.
2. S. K. Kim, S. Y. Kim, J. H. Kim, S. A. Roh, D. H. Cho, Y. S. Kim, J. C. Kim, A nineteen gene-based risk score classifier predicts prognosis of colorectal cancer patients. *Mol Oncol* **8**, 1653-1666 (2014).
3. A. McKenna, M. Hanna, E. Banks, A. Sivachenko, K. Cibulskis, A. Kernytsky, K. Garimella, D. Altshuler, S. Gabriel, M. Daly, M. A. DePristo, The Genome Analysis Toolkit: a MapReduce framework for analyzing next-generation DNA sequencing data. *Genome Res* **20**, 1297-1303 (2010).
4. G. A. V. d. A. B. D. O’Connor, "Genomics in the Cloud: Using Docker, GATK, and WDL in Terra" (O'Reilly Media, Sebastopol, CA, 2020).
5. B. Bushnell. (Joint Genome Institute, 2014).
6. M. M. Vasimuddin, S.; Li, H.; Aluru, S., paper presented at the 2019 IEEE International Parallel and Distributed Processing Symposium (IPDPS), Rio de Janeiro, Brazil, 2019.
7. P. Danecek, J. K. Bonfield, J. Liddle, J. Marshall, V. Ohan, M. O. Pollard, A. Whitwham, T. Keane, S. A. McCarthy, R. M. Davies, H. Li, Twelve years of SAMtools and BCFtools. *Gigascience* **10**, (2021).
8. A. Mayakonda, D. C. Lin, Y. Assenov, C. Plass, H. P. Koeffler, Maftools: efficient and comprehensive analysis of somatic variants in cancer. *Genome Res* **28**, 1747-1756 (2018).

9. K. Pelka, M. Hofree, J. H. Chen, S. Sarkizova, J. D. Pirl, V. Jorgji, A. Bejnood, D. Dionne, W. H. Ge, K. H. Xu, S. X. Chao, D. R. Zollinger, D. J. Lieb, J. W. Reeves, C. A. Fuhrman, M. L. Hoang, T. Delorey, L. T. Nguyen, J. Waldman, M. Klapholz, I. Wakiro, O. Cohen, J. Albers, C. S. Smillie, M. S. Cuoco, J. Wu, M. J. Su, J. Yeung, B. Vijaykumar, A. M. Magnuson, N. Asinovski, T. Moll, M. N. Goder-Reiser, A. S. Applebaum, L. K. Brais, L. K. DelloStritto, S. L. Denning, S. T. Phillips, E. K. Hill, J. K. Meehan, D. T. Frederick, T. Sharova, A. Kanodia, E. Z. Todres, J. Jane-Valbuena, M. Biton, B. Izar, C. D. Lambden, T. E. Clancy, R. Bleday, N. Melnitchouk, J. Irani, H. Kunitake, D. L. Berger, A. Srivastava, J. L. Hornick, S. Ogino, A. Rotem, S. Vigneau, B. E. Johnson, R. B. Corcoran, A. H. Sharpe, V. K. Kuchroo, K. Ng, M. Giannakis, L. T. Nieman, G. M. Boland, A. J. Aguirre, A. C. Anderson, O. Rozenblatt-Rosen, A. Regev, N. Hacohen, Spatially organized multicellular immune hubs in human colorectal cancer. *Cell* **184**, 4734-4752 e4720 (2021).
