# Supplementary material for: LIN28B-mediated PI3K/AKT pathway activation promotes metastasis in colorectal cancer models
Source: J Clin Invest. 2025 Jan 14;135(8):e186035. doi: 10.1172/JCI186035 (PMC11996871; doi:10.1172/JCI186035)

Full unedited blot/gel for Figure 1A

LIN28B

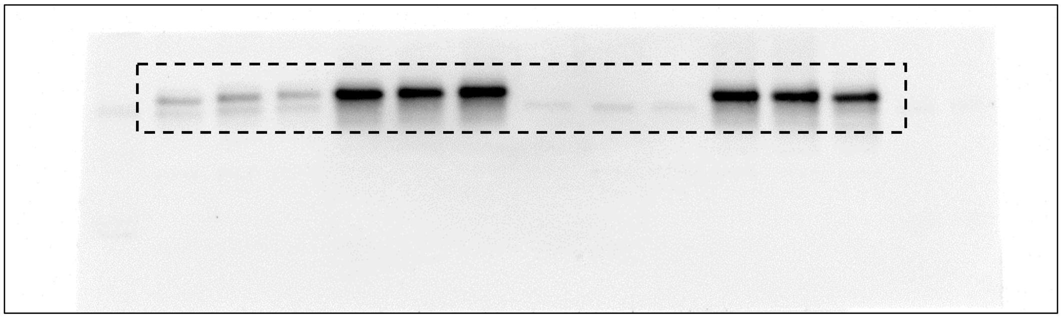

GAPDH

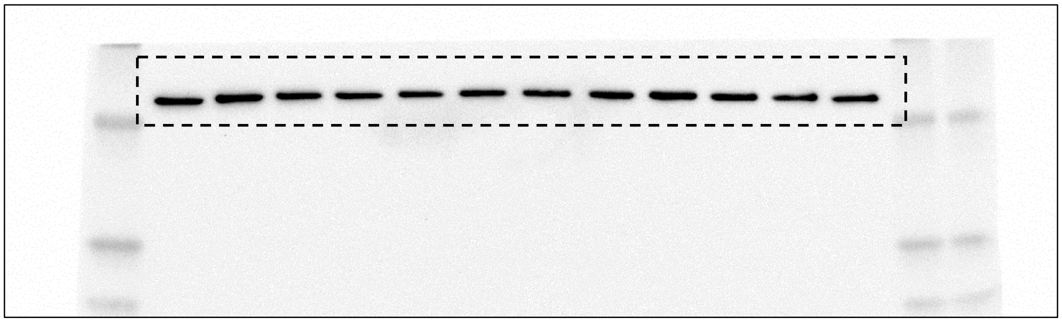

Full unedited blot/gel for Figure 4A

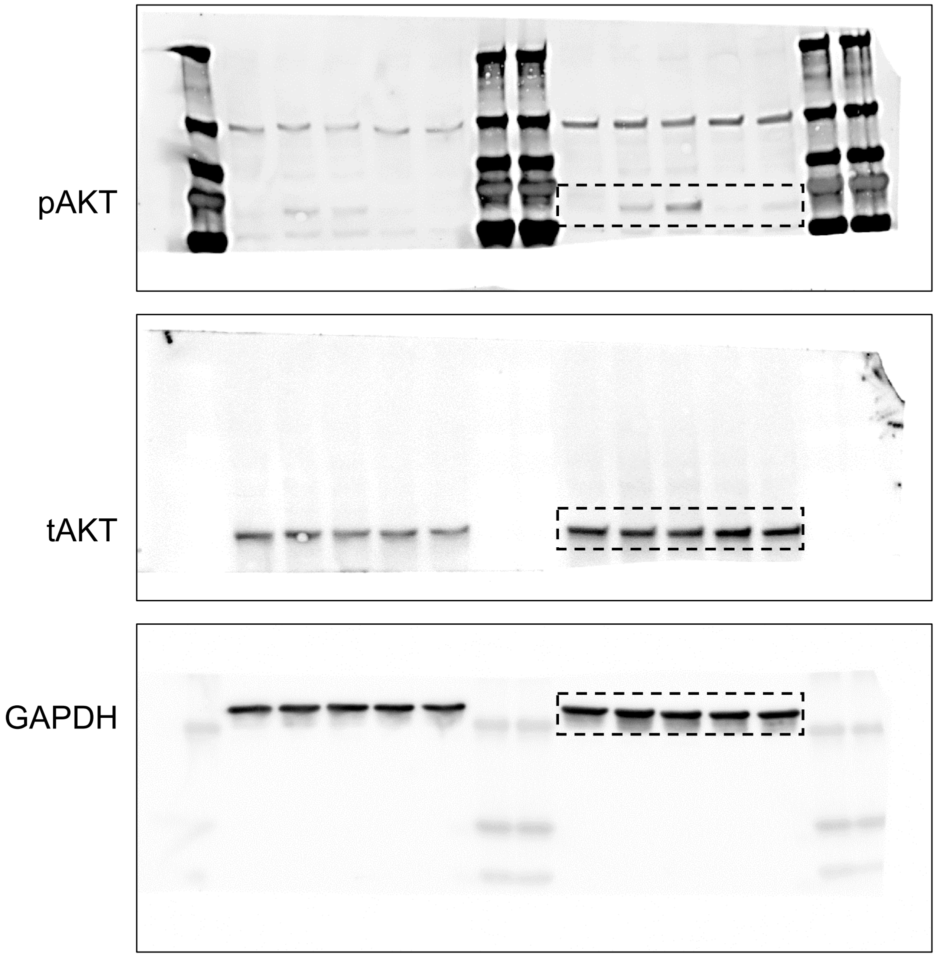

Full unedited blot/gel for Figure 4F

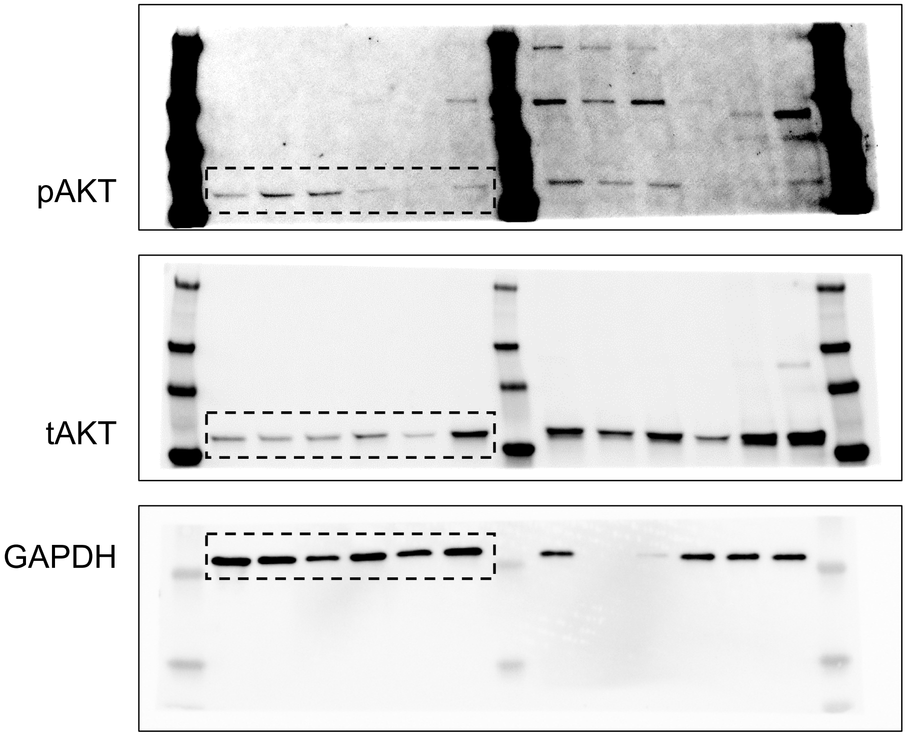

Full unedited blot/gel for Figure 6D

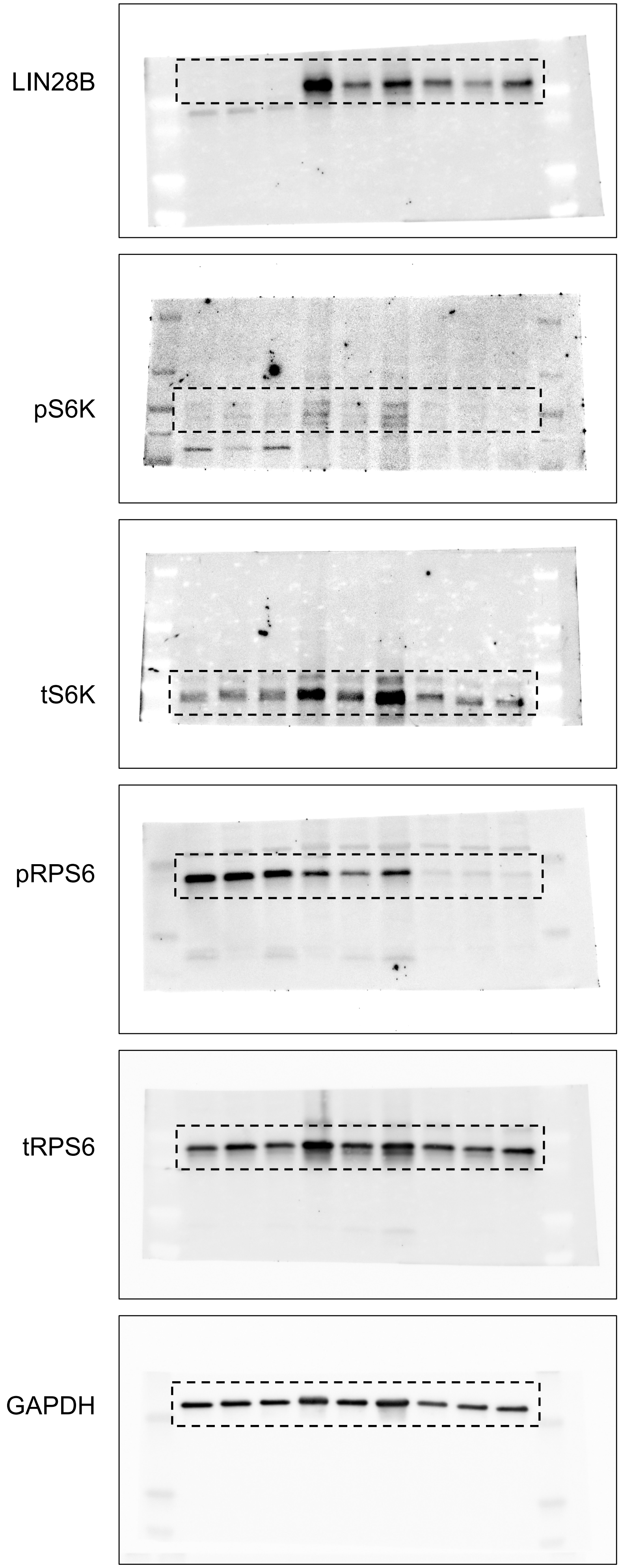

Full unedited blot/gel for Figure 6F

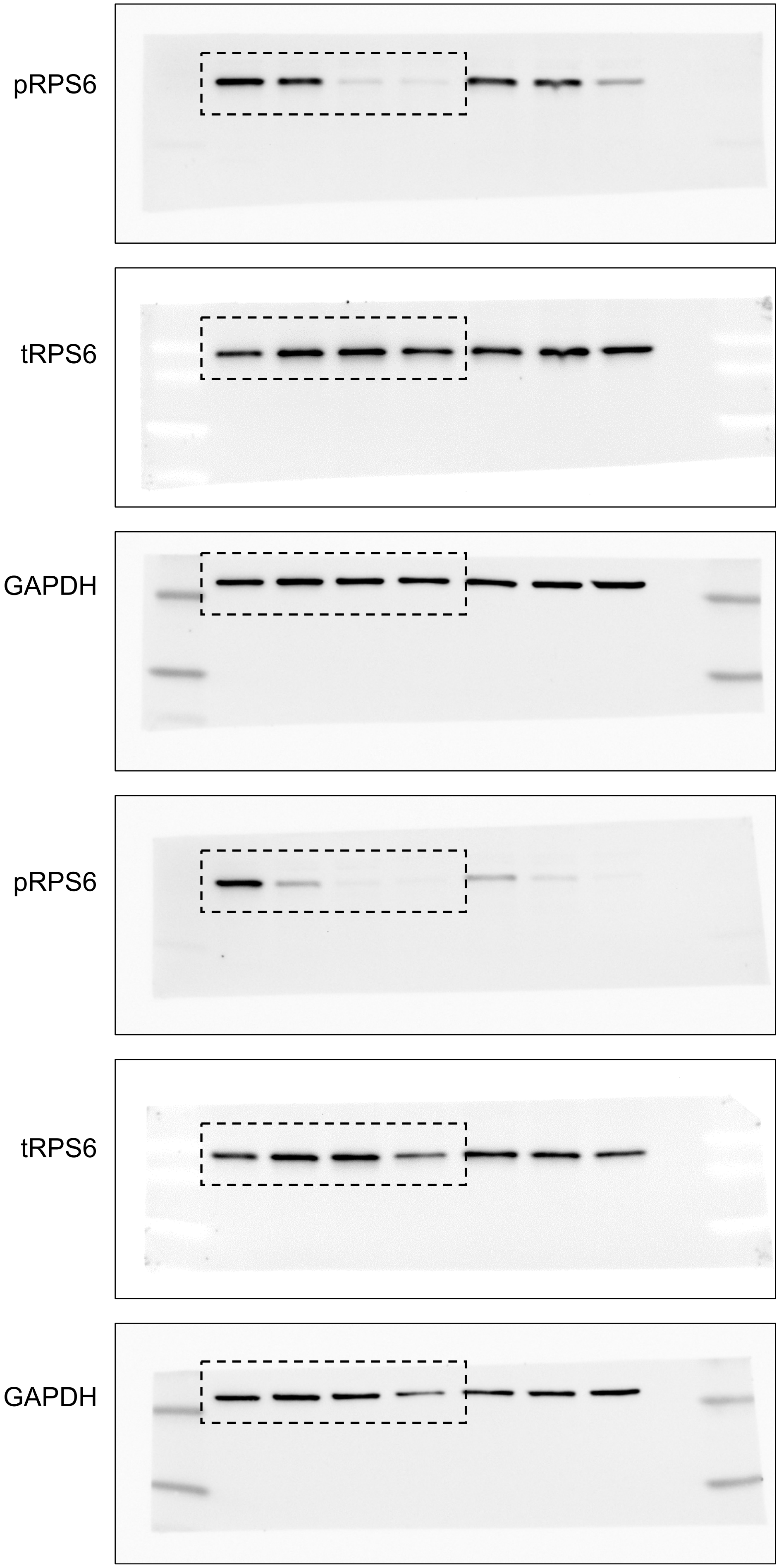

Full unedited blot/gel for Supplemental Figure 9C

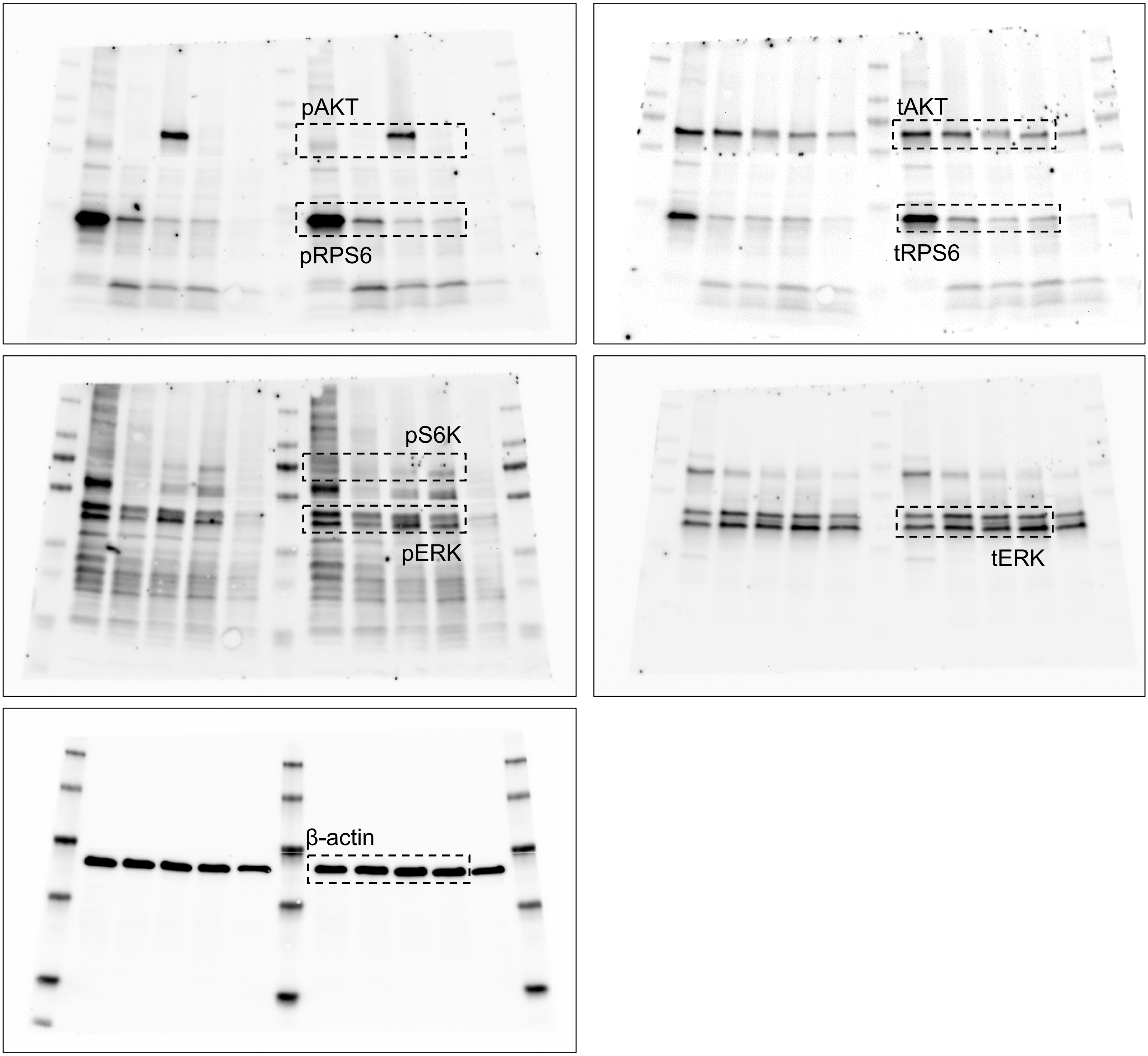

Supplement: Unedited blot and gel images [file jci-135-186035-s250.pdf]
